# Multiocular organoids from human induced pluripotent stem cells displayed retinal, corneal, and retinal pigment epithelium lineages

**DOI:** 10.1186/s13287-021-02651-9

**Published:** 2021-11-22

**Authors:** Helena Isla-Magrané, Anna Veiga, José García-Arumí, Anna Duarri

**Affiliations:** 1 Ophthalmology Research Group, Vall d’Hebron Institut de Recerca (VHIR), Vall d’Hebron Barcelona Hospital Campus, Passeig Vall d’Hebron 119-129, 08035 Barcelona, Spain; 2 Regenerative Medicine Program IDIBELL, L’Hospitalet de Llobregat, Barcelona, Spain; 3 Department of Ophthalmology, Vall d’Hebron Hospital Universitari, Barcelona, Spain; 4 Department of Ophthalmology, Universitat Autònoma de Barcelona, Bellaterra, Spain

**Keywords:** Stem cells, Ocular precursor cells, Ocular organoids, Retina, Cornea, RPE, Retinal pigment epithelium

## Abstract

**Background:**

Recently, great efforts have been made to design protocols for obtaining ocular cells from human stem cells to model diseases or for regenerative purposes. Current protocols generally focus on isolating retinal cells, retinal pigment epithelium (RPE), or corneal cells and fail to recapitulate the complexity of the tissue during eye development. Here, the generation of more advanced in vitro multiocular organoids from human induced pluripotent stem cells (hiPSCs) is demonstrated.

**Methods:**

A 2-step method was established to first obtain self-organized multizone ocular progenitor cells (mzOPCs) from 2D hiPSC cultures within three weeks. Then, after the cells were manually isolated and grown in suspension, 3D multiocular organoids were generated to model important cellular features of developing eyes.

**Results:**

In the 2D culture, self-formed mzOPCs spanned the neuroectoderm, surface ectoderm, neural crest, and RPE, mimicking early stages of eye development. After lifting, mzOPCs developed into different 3D multiocular organoids composed of multiple cell lineages including RPE, retina, and cornea, and interactions between the different cell types and regions of the eye system were observed. Within these organoids, the retinal regions exhibited correct layering and contained all major retinal cell subtypes as well as retinal morphological cues, whereas the corneal regions closely resembled the transparent ocular-surface epithelium and contained of corneal, limbal, and conjunctival epithelial cells. The arrangement of RPE cells also formed organoids composed of polarized pigmented epithelial cells at the surface that were completely filled with collagen matrix.

**Conclusions:**

This approach clearly demonstrated the advantages of the combined 2D-3D construction tissue model as it provided a more ocular native-like cellular environment than that of previous models. In this complex preparations, multiocular organoids may be used to model the crosstalk between different cell types in eye development and disease.

**Graphical abstract:**

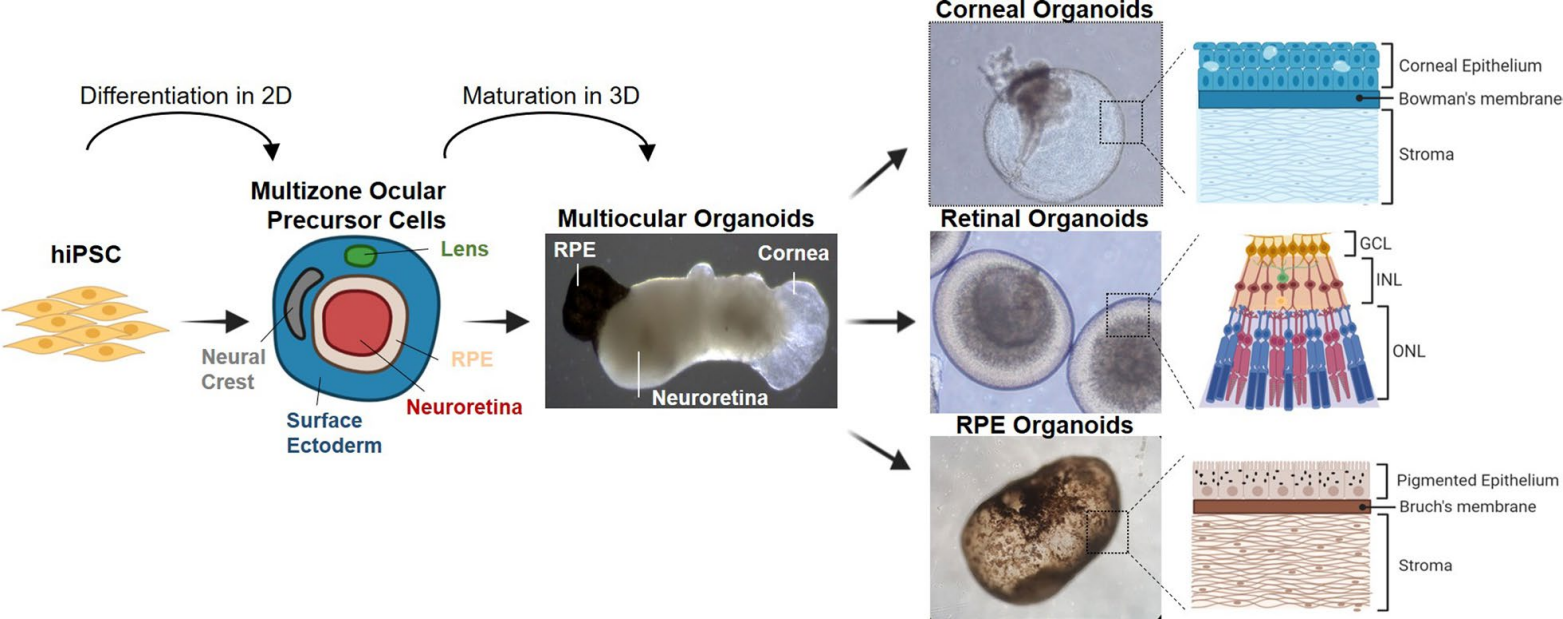

**Supplementary Information:**

The online version contains supplementary material available at 10.1186/s13287-021-02651-9.

## Background

The eye is a complex organ comprising different specialized ocular tissues that together are responsible for vision. Diseases or injuries affecting ocular tissues are causing increasing global problems, as they cause partial or total vision loss and affect approximately 2.2 billion people worldwide [1]. The eye is divided into anterior and posterior segments. The posterior segment is composed of the retina, the retinal pigment epithelium (RPE) and the choroid. During eye development, the neuroretina (NR) (as well as the RPE) is derived from the anterior neuroectoderm, and retinal progenitor cells produce all types of retinal neurons (including photoreceptors, bipolar cells, amacrine cells, horizontal cells, and ganglion cells) and Müller glia in a sequential manner [2, 3]. Photoreceptors, the light sensing cells that are among the most important retinal neurons responsible for vision together with RPE, are responsible for visual function [4]. On the other hand, the anterior segment of the eye is composed of the cornea, conjunctiva, limbus, lens and iris, which are derived from the lens placode that arises from the surface ectoderm (SE). The cornea is the outer layer of the eye, is developed from the SE and controls and focuses the entry of light into the eye. This transparent layer is formed by a stratified epithelium in the outer section, a collagen-rich stroma that is maintained by keratocytes, and the endothelium in the inner part [5]. The conjunctiva covers the sclera and is composed of 3–5 layers of epithelium with superficial scattered goblet cells that produce important mucins for the tear film. The limbus limits the cornea and conjunctiva, and it contains stem cells that are key for preserving corneal integrity by renewing corneal epithelium (CE); these cells are used for regenerative purposes [6]. Moreover, the neural crest (NC)-mesenchymal ectoderm-derived component contributes to from the corneal stroma and endothelium, ciliary body, and melanocytes [7].

Currently, much effort has been made to regenerate damaged or diseased retinas, RPE, and corneas by utilizing cell-based therapies to restore visual function. Since the discovery of human embryonic stem cells (hESCs) [8] and induced pluripotent stem cells (hiPSCs) [9], disease modeling, organoids, and cell therapy testing have contributed to finding promising therapeutic strategies to treat ocular diseases [10]. This is particularly interesting for studies involving eyes, which are composed of different highly specialized tissues derived from various cell linages, including neuroectoderm, SE, NC and the periocular mesenchyme [11]. hESC and hiPSC-derived ocular organoids have been described to endogenously recapitulate the architecture and cellular organization, and function of native ocular embryonic tissues [12]; this occurs because higher and more natural complexities are achieved and because they allow the generation of clinically relevant cell numbers and ratios, including their own stroma [13–16]. These ocular organoids are useful for regenerating ocular tissue by generating appropriate RPE [17], retinal [18–28], limbal [15] and corneal [29] cells for transplant therapies, and also could be used for modeling diseases involving both anterior and posterior parts of the eye, such as anophthalmia, microphthalmia, and ocular malformations, or rare ocular diseases of the neural crest caused by mutations in the eye-field transcription factors *SOX2*, *RAX*, *PAX6, PAX3*, *SOX10* and *CHX10* [30–34].

New methodologies to produce highly organized well-delimited zones of ocular tissues, such as retinal, corneal, limbal, conjunctival, RPE and lens progenitor cells have recently been developed [29, 35–38]. The self-formed ectodermal autonomous multizone (SEAM) of ocular cells from hiPSCs in a two-dimensional (2D) culture approach better emulates the complexities of ocular morphogenesis and might be a promising source for different ocular cells; however, current experimental designs only focus on the isolation of a single cell type, such as retinal or corneal cells, and they cannot recreate the intricate 3D ocular organization [29, 36, 38]. Moreover, the processes of generating conventional ocular-like tissue, which are crucial for multilineage communication and are required for tissue development, remains incomplete as they lack stromal components. Accordingly, strategies to improve the high-level organization of complex 3D ocular tissue, including the crosstalk between different ocular structures, are still needed.

Here, hiPSCs-derived mutizone ocular progenitor cultures in 2D were used to develop 3D multiocular organoids containing a combination of retina, cornea, conjunctiva, and RPE regions, and together with keratocytes, they produced stromal-like tissue (including collagen type I and IV). The 3D ocular organoids obtained possess the unique advantage of recapitulating the multiregional cytoarchitecture seen in early human eye development. Although multiocular organoids do not display native ocular tissue organization, they more closely model the important cellular features of developing eyes.

## Methods

### Human induced pluripotent stem cell culture

Two hiPSC lines were used in this study. The human cord blood-derived iPSC line CBiPS30-4F-5 (CB30) [39] and the fibroblast-derived iPSC line FiPS-4F-7 (FiPS) [40] were obtained from the Spanish National Stem Cell Bank after approval by the Ethics Review Board and the Catalan Authority for Stem Cells. Both iPSC lines were cultured and expanded in Matrigel-coated (Corning, NY, USA) plates in mTeSR1 medium (StemCell Technologies, Vancouver, BC, Canada). The cells were passaged with 1 mM EDTA at a ratio of 1:4–1:6 every 5–7 days, and the medium was changed every day. The cells were incubated at 37 °C in a humidified atmosphere containing 5% CO_2_.

### Differentiation of hiPSCs into multizone ocular progenitor cells

**Fig. 1:**
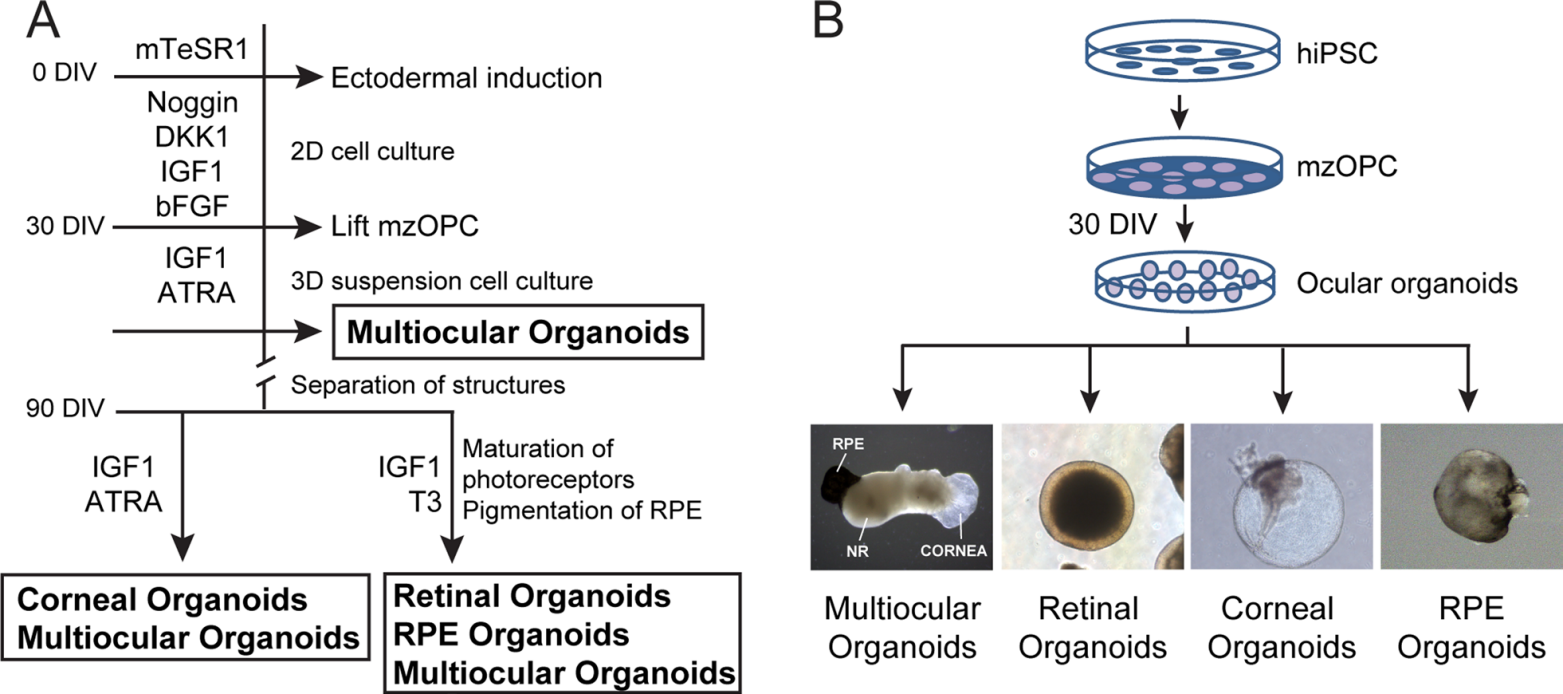
Schematic representation of the hiPSC differentiation protocol for multizone ocular progenitor cells and multiocular organoids. **A**, **B** Schemes of the differentiation protocol for the 2D generation of multizone ocular progenitor cells (mzOPCs) consisting of neuroectoderm, surface ectoderm and neural crest structures. At day 30, mzOPCs were manually picked and developed in suspension into 3D multiocular, retinal, corneal, and RPE organoids. Abbreviations: ATRA, all-trans retinoic acid; bFGF, basic fibroblast growth factor; DIV, days in vitro; DKK1, Dickkopf WNT Signaling Pathway Inhibitor 1; hiPSC, human induced pluripotent stem cells; IGF1, insulin-like growth factor 1; T3, triiodothyronine; RPE, retinal pigment epithelium

The differentiation protocol is depicted in Fig. 1 and was previously described [40]. Briefly, eye-field induction was performed using 75% confluent hiPSCs cultured on Matrigel (Corning, NY, USA) in induction medium (IM) consisting of Dulbecco’s modified Eagle’s medium/nutrient mixture F-12 (DMEM/F12), 5% fetal bovine serum, 0.1 mM nonessential amino acids, 2 mM GlutaMax, 1% N2, 1% B27 (all the previous reagents were from Gibco, Thermo Fisher Scientific, Inc., Waltham, MA, USA), 10 mM β‐glycerol phosphate (Sigma-Aldrich, St. Louis, MO, USA), recombinant human IGF1 (10 ng/ml; R&D Systems, Minneapolis, MN, USA) and 10 mM nicotinamide (Sigma-Aldrich, St. Louis, MO, USA) supplemented with Noggin (10 ng/ml; Peprotech EC Ltd., London, UK), DKK1 (10 ng/ml; Sigma-Aldrich, St. Louis, MO, USA) and bFGF (10 ng/ml; Peprotech EC Ltd., London, UK). Medium was changed every other day for 30 days. At day 30, the cell culture consisted of multizone ocular progenitor cells (mzOPCs) forming the following ocular-specific structures: surface ectoderm, neural crest, lens, stromal cells, NR, and RPE.

### Generation of multiocular organoids and individual retinal, corneal and RPE organoids

At day 30, mzOPC cultures were manually picked under a microscope with Stripper® tips (Cooper Surgical, Trumbull, CT, USA) and cultured to generate multiocular organoids. All organoids were grown in low-attachment plate in the same dish with IM supplemented with all-trans retinoic acid (ATRA, 10 µM; Sigma-Aldrich, St. Louis, MO, USA) for 60 days. During this period, multiocular organoids were formed. Alternatively, individual retinal, corneal/conjunctival or RPE organoids could be generated or isolated from multiocular organoids (Fig. 1A, B). At day 90, multiocular organoids as well as RPE and retinal organoids were separated and cultured in IM supplemented with triiodothyronine (T3, 20 nM; Sigma-Aldrich, St. Louis, MO, USA) to allow photoreceptor maturation and RPE pigmentation to occur (Fig. 1A), whereas multiocular and corneal organoids were kept in ATRA in order for the corneal-like tissue to maintain transparency. IM was replenished every other day for 140 days. Moreover, RPE organoids were disaggregated in TrypLE Select (Gibco, Thermo Fisher Scientific, Inc., Waltham, MA, USA) and cultured on Matrigel-coated plates in IM as described [41–43] to obtain an RPE cell monolayer. Because the RPE organoids contained mostly RPE cells and collagen matrix, purifying the RPE cell culture was not necessary. The functional characterization of the RPE cell culture is described in the Additional file 1 [40].

### Histology and immunochemistry

The organoids and cells were fixed in 4% (w/v) paraformaldehyde (PFA; EMS, Hatfield, PA, USA) overnight at 4 °C and washed in phosphate saline buffer (PBS). The organoids were then embedded in paraffin and cut into 5 µm sections. For immunohistochemistry tests, paraffin sections were deparaffined, and antigens were retrieved using citrate buffer (pH 6) at 100 °C. Paraffin-embedded sections, pieces of organoids or cells were permeabilized and blocked in 1 × PBS containing 0.5% Triton X-100 (Sigma-Aldrich; St. Louis, MO, USA) and 6% donkey serum (Sigma-Aldrich; St. Louis, MO, USA) for 1 h at room temperature. Primary and secondary antibodies were diluted in PBS with 0.1% Triton X-100 and 6% donkey serum and incubated overnight at 4 °C or for 2 h at 37 °C, respectively. The primary antibodies are listed in Additional file 1: Table S1. Nuclei were stained with 4′,6-diamidino-2-phenylindol (DAPI). Confocal images were acquired using a DM6000 microscope (Leica Microsystems, Wetzlar, Germany) and Zeiss LSM 980 (Zeiss, Jena, Germany) and processed using ImageJ (ImageJ software; NIH, Bethesda, MD, USA) and Adobe Photoshop (Adobe Systems Inc., San Jose, CA, USA).

### Electron microscopy

Electron microscopy was performed as described [41]. Briefly, for transmission electron microscopy studies, hiPSC-derived retinal and corneal organoids were fixed with 4% PFA for 1 h at 4 °C followed by 2.5% (w/v) glutaraldehyde in 0.1 M cacodylate buffer pH 7.2–7.4 for 2 h at 4 °C. The samples postfixation was carried out in 1% (w/v) osmium tetroxide for 2 h at 4 °C. Then, dehydration was performed in an ascending ethanol series and the samples were embedded in epoxy resin (all reagents were from EMS, Hatfield, PA, USA). Ultrathin sections were examined on a JEOL JEM-1011 transmission electron microscope (JEOL, Tokyo, Japan). For the scanning electron microscopy examinations, hiPSC-derived retinal pigment epithelial cells were fixed and dehydrated as described above. The cells were dried with a critical point dryer and metalized, and they were examined using a JEOL JSM-6390LV scanning electron microscope (JEOL, Tokyo, Japan).

### Polymerase chain reaction (PCR) and real-time quantitative PCR (RT-qPCR)

Total messenger RNA was isolated from the cells and organoids using PureLink RNA Mini Kit (Invitrogen, Carlsbad, CA, USA), and 1 µg of mRNA was reverse transcribed with the High-Capacity cDNA Reverse-Transcription Kit (Applied Biosystems, Waltham, MA, USA) [44]. For standard PCR, cDNA (10 ng) was amplified using MyTaq Red DNA Polymerase (Bioline, London, UK) by a standard amplification program as follows: a hot start activation (95 °C for 5 min), denaturing step (95 °C for 30 s), annealing step (60 °C for 30 s) and extension step (72 °C for 30 s), which was repeated for 35 cycles. PCR products were visualized by 2% agarose gels. For RT-qPCR, cDNA (10 ng in triplicate) was amplified using a SYBR Green Master kit (Life Technologies, Carlsbad, CA, USA) following the manufacturer’s instructions [40]. RT-qPCR reactions were run on the ABI PRISM 7900HT platform (Applied Biosystems, Foster City, CA, USA), and cycle threshold (Ct) values were normalized using *GAPDH* as a housekeeping gene. Data were analyzed using the 2^−ΔCt^ method. The primer sequences are listed in Additional file 1: Table S2.

### Statistical analysis

Data are expressed as the mean ± standard deviation (SD) of at least three independent measurements. Statistical analysis was performed using GraphPad Prism software (GraphPad Software Inc., San Diego, CA, USA). An unpaired two-tailored Student’s t-test was applied to determine statistical significances between 2 groups. For more than 2 groups, one-way or two-way ANOVA with Tukey’s post hoc test and significant differences (*P* < 0.05) were utilized.

## Results

### Multizone ocular precursor cells organized into neuroectoderm, neural crest and surface ectoderm areas

**Fig. 2:**
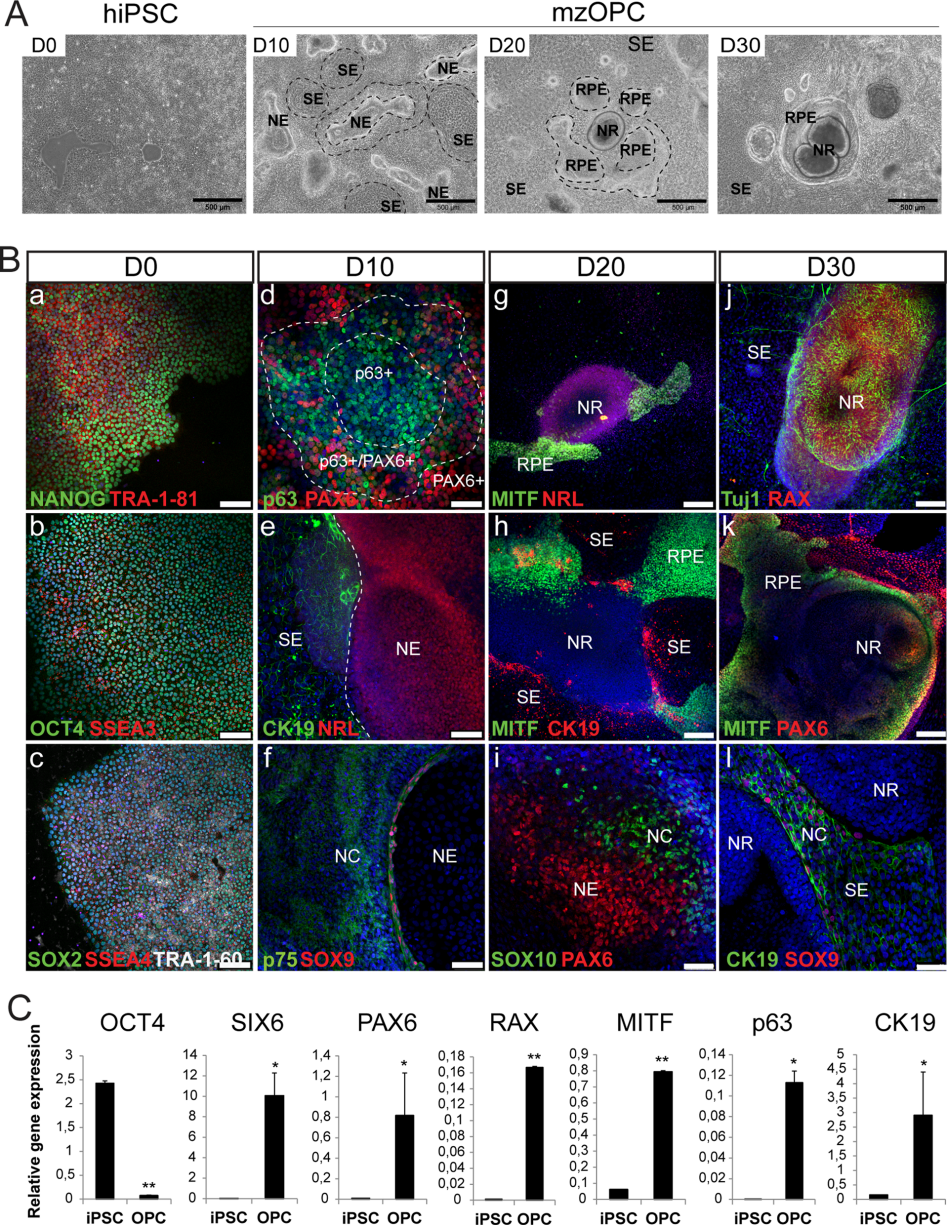
Multizone ocular progenitor cells expanded the neuroectoderm, surface ectoderm and neural crest cell regions. **A** Representative phase-contrast images of CB30 hiPSC differentiation toward multizone ocular progenitor cells (mzOPCs). The images of mzOPCs at 10 (D10), 20 (D20) and 30 (D30) DIV show the eye-field primordial clusters that develop in neuroectoderm (NE), surface ectoderm (SE), retinal pigment epithelium (RPE) and neural retina (NR) (*n* > 8 independent experiments). Scale bars: 500 µm. **B** Immunofluorescence images of hiPSCs expressing the pluripotency stem cell markers NANOG, TRA-1-81, OCT4, SSEA3, SOX2, SSEA4 and TRA-1-60 (a–c). mzOPCs at 10 DIV (d–f) and at day 20 (g–i) expressed NE-specific markers PAX6, NRL, and MITF; SE-specific markers CK19 and p63; and neural crest (NC)-markers SOX9, SOX10 and p75-NGFR. At day 30 (j–l), mzOPCs consisted of differentiated ocular clusters, including NR (RAX, TUJ1, PAX6); RPE (MITF); surface epithelial cells (CK19) and neural crest cells (SOX9). Nuclei were stained in DAPI. Scale bars: 100 µm in a–c, g, h, j,k; 50 µm in e, f,l; 25 µm in d, i. **C** Relative gene expression detected by RT-qPCR in hiPSCs and mzOPCs at 30 DIV of eye-field transcription factors *PAX6, RAX, SIX6*, SE markers *p63* and *CK19*, the RPE-specific marker *MITF,* and the pluripotency marker *OCT4.* Values are normalized to *GAPDH*. Data are presented as the mean ± SD (*n* = 3 independent experiments). Values indicated with stars are significantly different from those in hiPSCs (Student’s t-test; *p < 0.05; **p < 0.001)

Eye field commitment in the 2D hiPSC cultures was induced by inhibiting BMP and Wnt pathways using noggin and DKK1 antagonists, respectively [45], and by activating FGF and IGF-1 pathways [37, 46] (Fig. 1A). This led to the spontaneous formation of well-delimited neuroepithelium (NE) and surface ectoderm (SE) zones beginning at 10 days in vitro (DIV), and they continued to form until 30 DIV in both the CB30 (Fig. 2; Additional file 1: Figure S1) and FiPS (Additional file 1: Figure S2) cell lines. During the differentiation procedure, multizone ocular precursor cells (mzOPCs) self-formed a concentric arrangement with distinct cell morphologies (Fig. 2A; Additional file 1: Figures S1A-B and S2A), which was similar to SEAM protocols [29, 35, 36]. The innermost zone corresponded to the well-formed NR with a characteristically round shape, and it had clear margins and an early laminated apical layer containing retinal precursor cells that expressed PAX6, TUJ1, CHX10 and RAX (Fig. 2A, B; Additional file 1: Figures S1C and S2B). The second zone, corresponded to RPE cells that expressed MITF. And in the outermost zone, the SE contained cells that expressed CK19 and p63, lentoid cluster cells that expressed γ-crystallin, and stroma-like cells that expressed vimentin (VIM). Neural crest (NC) cells expressing SOX9, p75-NGFR and SOX10 were also present in the SE close to the NR (Fig. 2B; Additional file 1: Figures S1A-C and S2B). Similarly, mzOPCs expressed the eye-field transcription factors *PAX6, RAX, SIX3, SIX6* and *LHX2*, the NR-specific *CHX10,* the photoreceptor-specific *CRX*, SE markers *p63* and *CK19*, the RPE-specific marker *MITF,* and NC markers *PAX3*, *SOX9* and *TFAP2A.* The pluripotency marker *OCT4* was not expressed at this stage (Fig. 2C; Additional file 1: Figures S2C, S5C and S6A). These results increase our understanding of embryogenesis and provide a method by which the relative induction of eye-field-committed precursor cells that span the RPE, retinal, SE, NC, lens, and stromal cells can be modulated. From here, mzOPC clusters were manually isolated in an attempt to generate free-floating 3D multiocular organoids.

### Multiocular organoids exhibited neuroretinal, pigmented epithelium and corneal-conjunctival regions

**Fig. 3:**
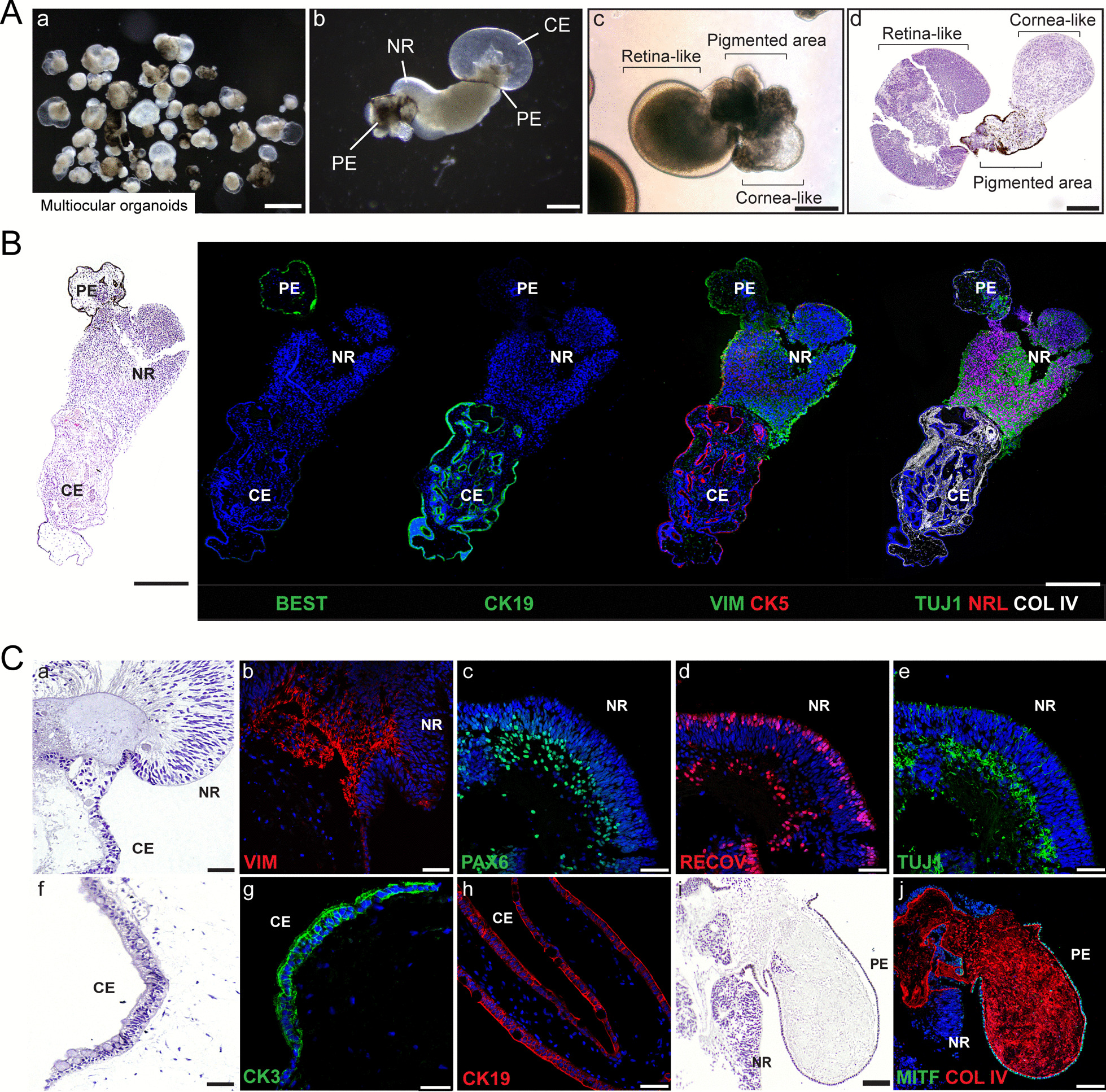
Multiocular organoids were composed of neuroretina, pigmented region and corneal-like epithelium and endothelium, and conjunctiva. **A** Bright field and hematoxylin and eosin (HE) images of CB30-derived multiocular organoids at 90 DIV showing the following different ocular regions: neuroretina, pigmented area, and corneal-like. Scale bars: 3 mm (left image); 500 µm in bright field; 250 µm in HE. **B** HE and confocal images of representative multiocular organoid paraffin sections at 90 DIV consisting of pigmented epithelium (PE; bestrophin-1 (BEST)), corneal epithelium (CE; CK19 and CK5), neuroretina (NR; NRL and TUJ1), stromal cells (vimentin (VIM)), and collagen type IV (COL IV)). Scale bars: 500 µm. **C** HE (a, f, i) and confocal images of detailed ocular areas. NR expressed VIM, PAX6 and TUJ1 in the inner layer and recoverin (RECOV) in the outer layer (a–e), CE expressed CK3 (corneal marker) and CK19 (conjunctival marker) (g, h), and PE expressed MITF (RPE marker) and COL IV. Scale bars: 50 µm. Nuclei were stained with DAPI

**Fig. 4:**
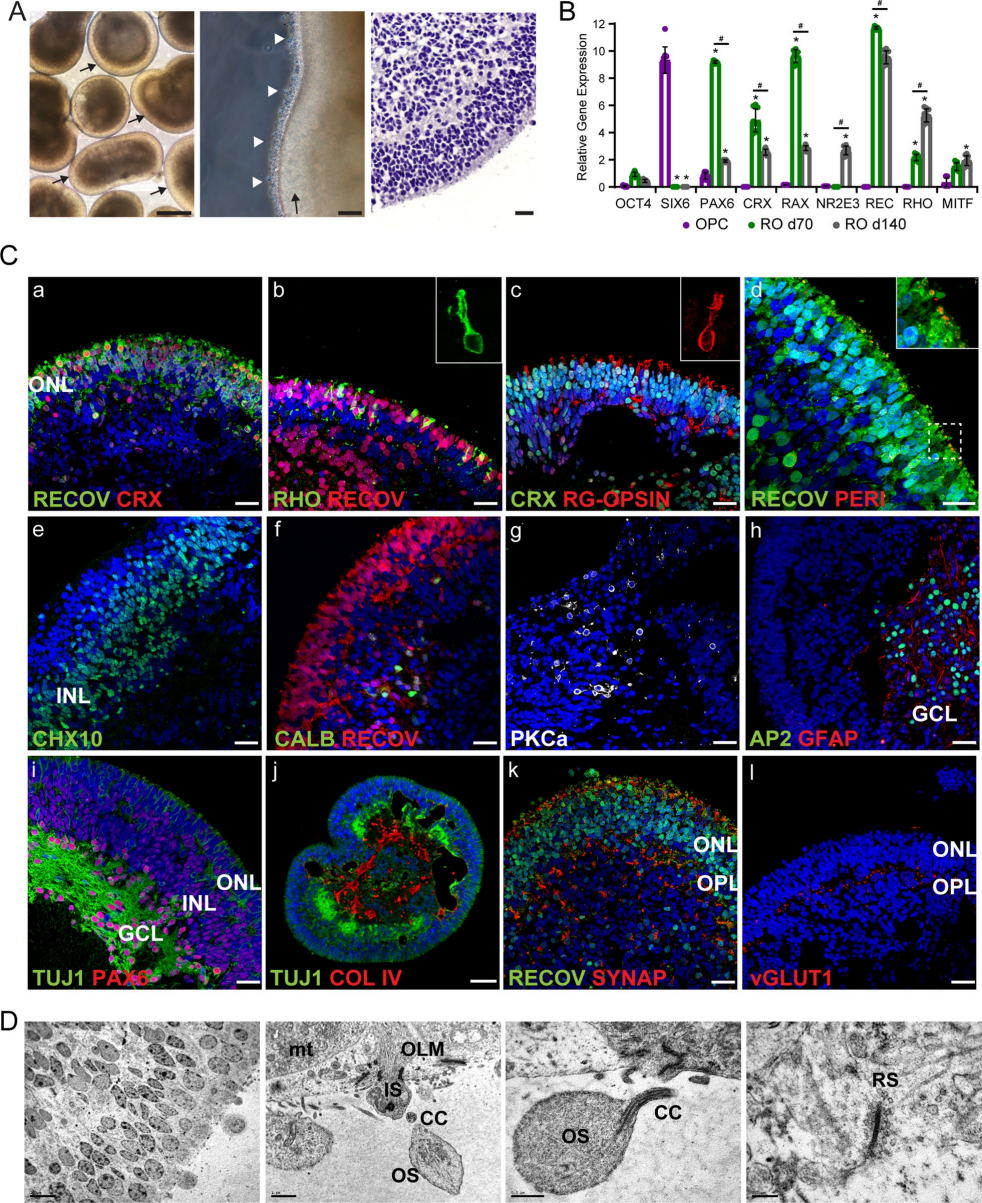
Development and organization of the retinal layer within retinal organoids generated from CB30-derived mzOPCs. **A** (Left-middle) Bright-field images of retinal organoids derived from the CB30 hiPSC line at 140 DIV. The arrows indicate developing retinal tissue, and arrowheads indicate developing photoreceptor inner/outer segments. (Right) Hematoxylin and eosin staining of paraffin sections showing lamination of retinal tissue. Scale bars: 300 µm (left); 100 µm (middle); 50 µm (right). **B** RT-qPCR analysis of gene expression in mzOPCs at 30 DIV and retinal organoids at 70 (RO d70) and 140 DIV (RO d140). Values are normalized to *GAPDH*. Data are presented as the mean ± SD (*n* = 3 independent experiments). Two-way ANOVA and Tukey’s post hoc tests were performed, and significant differences are represented with *p < 0.001 vs. OPC; ^#^*p* < 0.001 RO d70 vs. RO d140. **C** Confocal images of retinal organoid paraffin sections at 140 DIV immunostained with photoreceptor cell markers CRX, RECOV, rhodopsin (RHO), RG-opsin, and pericentrin (PERI) (a–d) corresponding to the outer nuclear layer (ONL); bipolar cell markers CHX10 and PKC-alpha and horizontal cell markers CALB (calbindin-28 K) (e–g) corresponding to the inner nuclear layer (INL); ganglion cell markers TUJ1 and PAX6 (i, j) corresponding to the ganglion cell layer (GCL); and synaptic markers SYNAP (synaptophysin) and vGLUT1 (k, l) corresponding to the outer plexiform layer (OPL). Scale bars: 100 µm in I, j; 25 µm in a–h, k, l. **D** Transmission electron microscopy analysis of retinal organoids at 140 DIV shows several retinal structures of connecting cilia (CC), inner segments (IS), mitochondria (mt), outer limiting membrane (OLM), outer segments (OS) and ribbon synapses (RS). Scale bars: 10 µm (a, b); 2 µm (c); 1 µm (d); 0.5 µm (e, f, g); 0.2 µm (h)

**Fig. 5:**
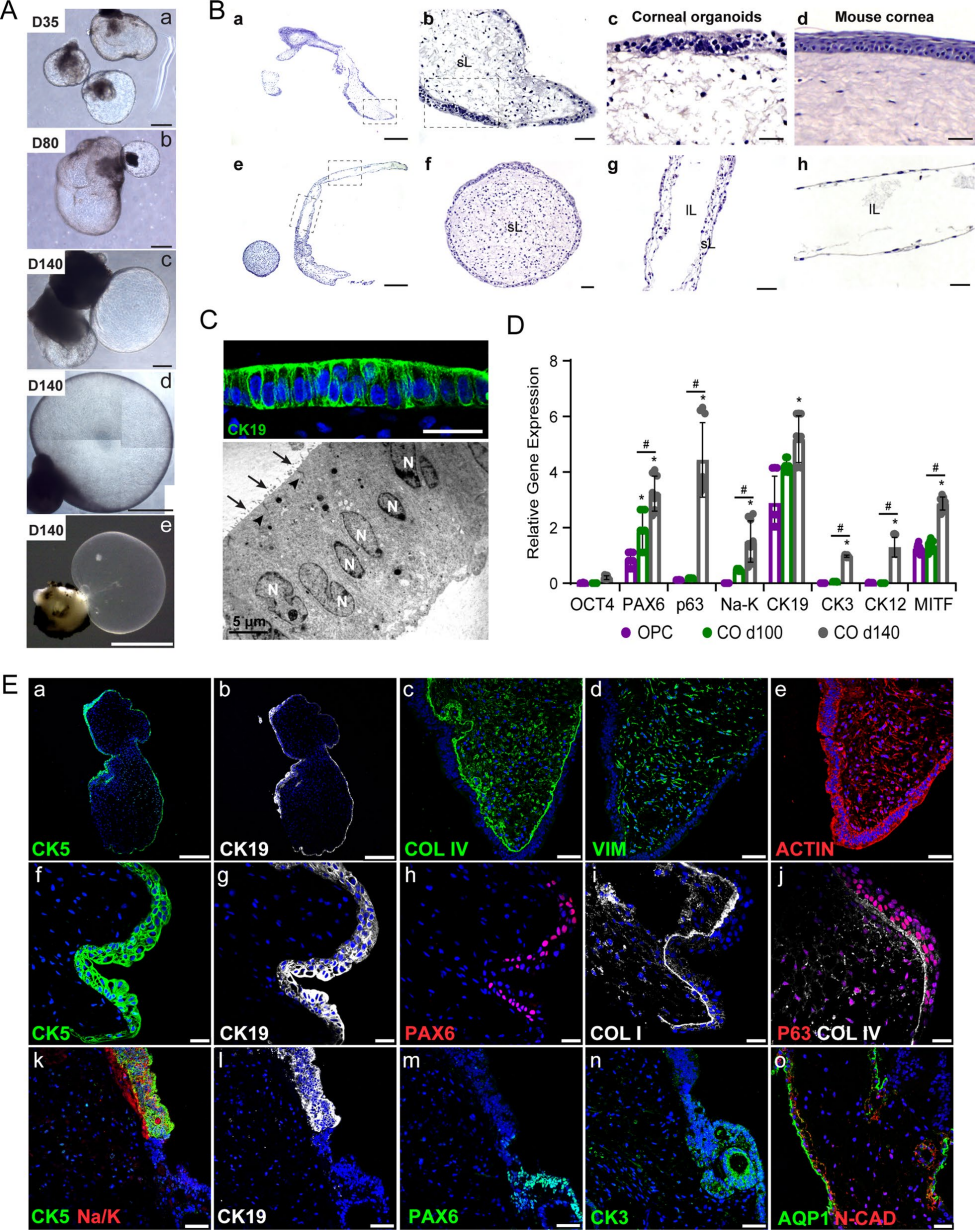
Corneal organoids exhibited areas of conjunctiva and areas of CE and endothelium. **A** Bright-field images of developing corneal-like organoids derived from CB30 hiPSCs cultured for 35 (D35), 80 (D80), and 140 (D140) DIV. Scale bar: 300 µm in a–c; 1 mm in d; 2 mm in e. **B** Hematoxylin and eosin staining of corneal organoids at 140 DIV. Corneal organoids with stromal lumen (a, b, f). The dashed squared in (a) indicates magnified image in (b). Stratified surface epithelium (c) with collagen-filled stroma resembling native mouse cornea (d). Corneal organoids with fluid-filled stroma and an endothelium-like layer (e, g, h). Dashed squares in (e) indicate magnified images in (g, h). Scale bars: 300 µm in a, e; 50 µm in b, f–h; 25 µm in c, d. **C** Immunohistochemistry of the surface epithelium stained with cytokeratin 19 (CK19) (top panel). Scale bar: 10 µm. Transmission electron microscopy image of corneal organoids at 140 DIV showing corneal epithelial cells with apical microvilli (arrows), tight junctions between cells (arrowheads) and cell nuclei (N) (bottom panel). Scale bar: 5 µm. **D** RT-qPCR analysis of gene expression in mzOPCs at 30 DIV and in corneal organoids at 100 (CO d100) and 140 DIV (CO d140). Values were normalized to *GAPDH*. Data are presented as the mean ± SD (*n* = 3 independent experiments). Two-way ANOVA and Tukey’s post hoc tests were performed, and significant differences are represented with *p < 0.001 vs. OPC; ^#^*p* < 0.001 CO d100 vs. CO d140. **E** Confocal images of immunostained corneal organoids at 140 DIV showing surface conjunctiva/limbal cells expressing CK5 (a, f, k), CK19 (b, g, l) and Na+/K+-ATPase (Na/K; k), corneal epithelial cells expressing PAX6 (h, m), cytokeratin 3 (CK3; n) and corneal endothelium expressing aquaporin-1 (AQP1) and N-CAD (o); and stroma containing cells positive for collagen type IV (COL IV; c, j), collagen type I (COL I; i) and vimentin (VIM) (d). The surface epithelium was delimited by COL IV and I, with p63 + cells on the apical side and some PAX6 + cells on the basal side (h, i, j). The cytoskeleton was stained with actin (e). Scale bars: 250 µm (a, b); 50 µm (c–e, k–n); 25 µm (f–j, o). Nuclei were stained with DAPI

**Fig. 6:**
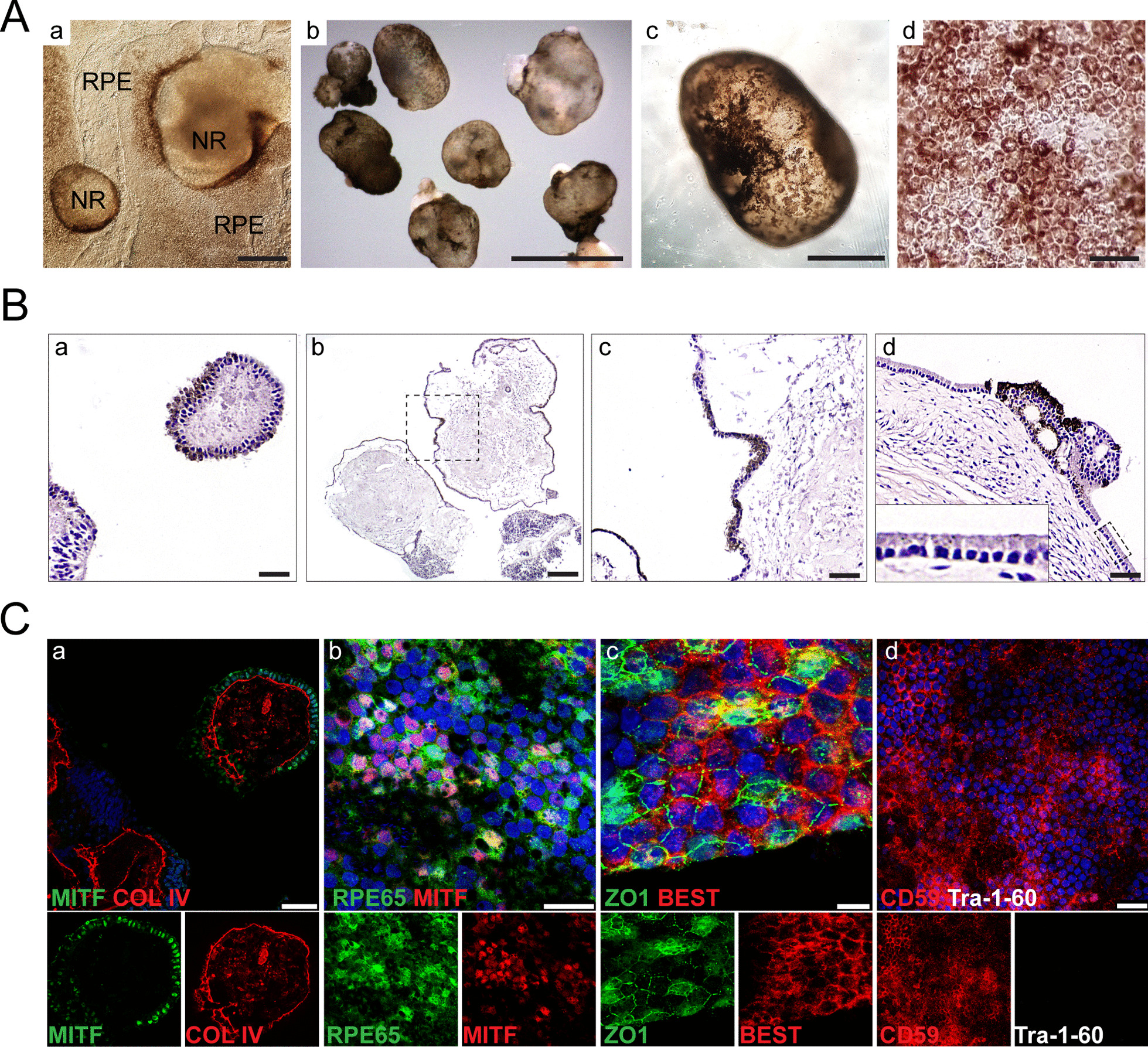
Retinal pigment epithelial organoids acquired pigmentation in the presence of T3 hormone. **A** (a) Phase contrast microscopy images of CB30-derived mzOPCs cultured for 30 days showing neuroretinal areas (NRs) surrounded by RPE cells. Scale bar: 250 µm. (b–d) Bright-field images of CB30-derived RPE organoids cultured in suspension for 120 days in the presence of T3 from day 90. Scale bars: 3 mm in b; 1 mm in c; 25 µm in d. **B** Hematoxylin and eosin staining of RPE organoid paraffin sections. The dashed square in b indicates the magnification area shown in c. Scale bars: 100 µm in b; 50 µm in a, c, d. **C** (a) Immunostaining of a paraffin section with antibodies against MITF and COL IV (**B**-a and **C**-a images correspond to the same sections). Confocal immunofluorescence images of the RPE organoid surface with the following RPE-specific markers: RPE65, MITF, ZO-1, BEST (bestrophin-1), and CD59 (b-d). Tra-1-60 was used to detect undifferentiated cells. Scale bars: 50 µm in a; 25 µm in b, d; 7.5 µm in c. Nuclei were stained with DAPI

Multiocular organoids were obtained from mzOPC and cultured in low-attachment plates (Figs. 1B, 3A; Additional file 1: Figure S3, S4 and S5). As a result, this process increased the proportion of large multiregion organoids in 3D culture that exhibited variable ocular tissues. The tissues were composed of multiple ocular identities, such as the following: (i) retina, RPE and cornea (Fig. 3; Additional file 1: Figures S3 and S4); (ii) retina and cornea (Additional file 1: Figure S4); (iii) retina and RPE (Fig. 3); and (iv) cornea and RPE (Additional file 1: Figure S5). Moreover, individual retinal, corneal, or RPE organoids were also obtained (Figs. 4, 5 and 6, respectively).

Analysis of large multiocular organoids (~ 1.5 mm) revealed that these organoids were formed by pigmented epithelium (BEST +), followed by NR (VIM + , NRL + and TUJ1 +) and a corneal-like region (CK5 + and CK19 +) (Fig. 3B). The internal corneal epithelium (CE) area was filled with stroma (COL IV). We then characterized different ocular tissues within the multiocular organoids in more detail. In the areas with laminated NR, the cells expressed PAX6 and TUJ1 filled with stromal cells (VIM) and collagen IV in the inner retinal layer and expressed recoverin and CRX corresponding to photoreceptor cells in the outer layer (Fig. 3C-a-e; Additional file 1: Figures S3, S4 and S5). The CE consisted of corneal epithelial cells (CK3), endothelium-like cells (AQP1 and N-CAD) (Fig. 3C-f-g; Additional file 1: Figure S3-n, o) [47] and conjunctival-limbal epithelium areas (CK19, CK5, Na+/K+-ATPase, SSEA1 and mucin-1) [6] filled with collagen type IV and stoma cells (VIM) (Fig. 3B and C-h; Additional file 1: Figure S3 and S4). Moreover, some areas showed a pigmented epithelium (PE) expressing MITF and bestrophin 1 (Fig. 3B and C-i, j; Additional file 1: Figure S3B and S5).

Individual organoids could be obtained from mzOPCs by isolating specific areas and also from multiocular organoids. To do this, we sectioned each part of these large organoids between days 60 and 90 and cultured them as a single retinal, RPE or corneal organoid (Figs. 4, 5 and 6). We calculated the efficiency of each type of organoid production, which was counted at day 90 of the differentiation processes beginning with the 75% confluent CB30 and FiPS cultures. From 1-well of a 6-well plate, we obtained 23.5 ± 4 multiocular or 53.3 ± 5 individual organoids (n = 5) from the CB30 line and 19.6 ± 7 multiocular or 56.6 ± 7 individual organoids (n = 4) from the FiPS line. The utility of stem cell-derived ocular organoids for disease modeling or drug screening depends upon the cellular and functional maturation of the different ocular cells within these organoids. To test this hypothesis, we focused on characterizing individual organoids.

### Neuroretinal structures produce 3D retinal organoids

Isolating NR areas produced retinal organoids that exhibited bright laminated retinal structures with a continuous cell layer, which was observed at 40 DIV by hematoxylin and eosin (HE) staining (Additional file 1: Figure S7A). The retinal progenitor cells in the retinal organoids coexpressed RAX and PAX6 and were scattered throughout the epithelium. Retinal cells at the apical side of the organoid expressed SOX2, NRL, CHX10, ZO-1 and recoverin, which corresponded to a developing outer nuclear layer (ONL); while TUJ1 staining was localized to the inner part of the structure, mimicking a developing ganglion cell layer (GCL) (Additional file 1: Figure S7B).

Retinal organoids were clearly larger and more mature at day 140 DIV and acquired a 3D-stratified retinal structure with a discrete apical layer (Fig. 4A). RT-qPCR analysis showed an increase in the expression of the photoreceptor-specific transcripts *CRX, RAX, LHX2, recoverin* and *rhodopsin* in retinal organoids at day 70, and it was maintained up to day 140. At both time points, *NR2E3*, *RHO* and *MITF* expression was increased and *SIX6* expression was decreased (Fig. 4B; Additional file 1: Figure S7C). Immunohistochemistry showed that cells within the well-organized ONL coexpressed the photoreceptor markers CRX, recoverin, rhodopsin and RG-opsin (Fig. 4C-a-c). Recoverin + cells harbored inner and outer segments (IS/OS) of photoreceptors on the apical surface expressing pericentrin in the connecting cilia (Fig. 4C-d). Cells in the INL expressed CHX10, PKCα and calbindin, although it was less evident, and these corresponded to bipolar and horizontal cells (Fig. 4C-e–g). In contrast, cells in the inner part of the organoid expressed GFAP, AP2, TUJ1 and PAX6, corresponding to ganglion cells in the developing GCL, and were surrounded and supported by extracellular matrix protein collagen IV (Fig. 4C-h-j). PAX6 exhibited a expression pattern that descended from the GCL toward the ONL (Fig. 4C-i), mimicking the developing human retina at 130 days of gestation [48]. The expression of synaptophysin and vGLUT1 demonstrated the synaptic connection between photoreceptors and secondary neurons in a developing outer plexiform layer (OPL) (Fig. 4C-k, l). Ultrastructural analysis of retinal organoids at 70 DIV by transmission electron microscopy showed partially segregated cells in the ONL that contained photoreceptor IS with mitochondria, outer limiting membrane, and photoreceptor OS structures containing basal bodies with protruding cilia and synaptic vesicles (Additional file 1: Figure S7D). At 140 DIV, the presence of photoreceptor-specific sensory cilia, including IS- and OS-containing intracellular membrane discs and photoreceptor ribbon synapses, corroborated their maturation (Fig. 4D).

### Surface ectoderm areas form 3D corneal organoids

Isolated SE zones consist of multipotent cells that can produce corneal organoids with multiple lineages, including CE, conjunctiva and limbus, NC-derived CE and stromal cells [5, 49]. Corneal organoids (organoids composed of CE, endothelium, or conjunctiva) were spherical with a translucent/transparent layer (Figs. 5A and 6B). Their size could be increased over time from 0.5 to 3 mm. At DIV 140, the paraffin section HE staining results revealed that the translucent organoids consisted of corneal/conjunctival-like stratified epithelium and lumen filled with stroma (Fig. 5B-a-f) that resembled the native corneal structure (Fig. 3B-compare c and d). The transparent organoids also consisted of simple squamous epithelium similar to the corneal endothelium-like membrane and fluid-filled lumen that collapsed after fixation (Fig. 5B-e, g, h). The TEM results revealed areas with 1–2 cell-thick layers that were formed by simple columnar cells expressing CK19, forming an epithelium with pronounced nucleoli rich in keratin filaments, tight junctions and apical microvilli, which is typical of transit-amplifying epithelial cells (Fig. 5C) [50]. Expression of conjunctival/limbal genes *p63* and *Na*+/*K*+*-ATPase,* corneal genes *CK12, CK3, PAX6, COL8A1, COL8A2 and AQP1,* and the RPE gene *MITF* in the corneal organoids was corroborated by PCR and qPCR (Fig. 5D; Additional file 1: Figure S8A)*.*

Immunohistochemistry analysis showed that several organoids contained conjunctival/limbal-like cell clusters expressing CK19, CK5 and p63, suggesting there was a primitive epithelial surface (Fig. 5E-a, b, f, g,j-l; Additional file 1: Figure S8B). We observed a more developed layer of stratified squamous cells containing 3–6 cell nuclei with slightly different cell shapes that depended on their basal or apical location (Fig. 5E-e-l). The internal lumen was filled with stromal keratocytes that expressed vimentin and deposited collagen types I and IV, which in turn formed a Bowman’s-like membrane (Fig. 5E-c, d, i, j; Additional file 1: Figure S8B and S8C). Bowman’s membrane maintained the corneal structural integrity of corneal organoids during maturation, which allowed it to have a spherical shape. In contrast, the fluid-filled stoma was surrounded by either Na+/K+-ATPase endothelial-like cells or by a thin layer of stromal cells that expressed aquaporin-1 and N-cadherin (Fig. 5E-o; Additional file 1: Figure S8C-e). Moreover, PAX6 expression was evident in the basal cells, whereas p63 + cells were present at the apical surface (Fig. 5E-h, j). In some areas, there was a clear transition between conjunctival-like epithelia (CK19 + , CK5 + , Na^+^/K^+^-ATPase + , p63 + cell niche) and corneal-like epithelium (PAX6 + and CK3 +) (Fig. 5E-k–n; Additional file 1: Figure S6B and S8B). Interestingly, a few pigmented cells were also observed at the periphery of some organoids (Additional file 1: Figure S8B-g), indicating the potential presence of ciliary margin zone-like pigmented cells derived from nonneural progenitors, which contribute to stromal cell infiltration and ocular-surface formation [38, 51, 52].

### RPE organoids acquired polarization and pigmentation

RPE organoids were obtained either by isolating the RPE cell regions surrounding the NR in mzOPCs or by separating RPE regions from multiocular organoids (Fig. 6A; Additional file 1: Figure S6C and S9), similar to the report by Liu et al. [53] and Susaimanickam et al. [38]. After removing ATRA at day 90 in culture, RPE organoids acquired pigmentation (Fig. 6A-b-d; Additional file 1: Figure S9A). RPE cells at the periphery of the organoids formed a cell monolayer with apical polarization toward the outermost part of the organoid, and in the basolateral site, RPE cells produced considerable collagen IV, which is a component of the underlying Bruch’s membrane, and filled the organoid lumen (Fig. 6B and C-a; Additional file 1: Figure S6C-c). RPE cells in these organoids expressed RPE65, MITF, ZO1, bestrophin, CD59, and CD140, but not Tra-1–60, a marker of pluripotency (Fig. 6C-b-c; Additional file 1: Figure S6C-d-f). From these organoids, RPE cells could be isolated and cultures as a cell monolayer. The resulting RPE monolayer expressed PAX6, MITF, bestrophin, CHX10 and ZO-1 (Additional file 1: Figure S9B). RPE cell culture exhibited the typical hexagonal cell shape with apical microvilli, melanin-containing melanosomes and tight junctions between cells (Additional file 1: Figure S9C). Pigmentation of RPE cells was coincident with functional maturation. To assess whether the RPE organoids and isolated RPE cells retained correct functionality in vitro, we tested their phagocytosis capacity and barrier functions. Cultured RPE cells phagocytosed FITC-labeled photoreceptor outer segments that appeared in the cytoplasm (Additional file 1: Figure S9D). Moreover, the epithelial barrier of RPE in culture, which was measured by TEER, exhibited an increased resistance after 3 weeks and reached 250 Ώ·cm^2^ (Additional file 1: Figure S9E). These results suggested that RPE cells derived from RPE organoids exhibited mature cell morphology and function.

## Discussion

While tremendous progress has been made in understanding the highly complex mechanisms involved in eye-field differentiation, current in vitro protocols fail to emulate the spatial cellular organization of the eye. Most differentiation protocols using hiPSC-derived ocular cells and organoids focus on a single tissue-specific lineage, which fail to reflect the tissue complexity of eye development. With the aim of assessing how different ocular cell tissues organize and interact with each other within the same organoid, we demonstrated the remarkable ability of multiocular organoids to self-organize from mzOPCs in suspension and to form distinct eye structures, such as the retina, cornea and RPE, which are interconnected.

The aim of this study was to obtain an organoid system model that could generate complex ocular organoids from the same hiPSC culture, or basically from the same individual. It is necessary to employ all types of ocular progenitors to proceed with the formation of multiocular organoids by self-arrangement, but they have not been obtained altogether using embryoid bodies-based protocols yet. The reason for initiating differentiation from a 2D culture system is that 3D systems are not that effective in generating anterior eye tissue such as the cornea, although they have shown a high capacity to develop retinal organoids [54]. Therefore, for the initial induction of differentiation, we used a 2D culture approach to generate mzOPCs for the early stage of eye-field differentiation based on our [40] and previous studies on SEAM ocular cells [29, 35, 36, 40, 55] with some modifications. The differentiation started with 75% confluent hiPSC cultures instead of single colonies, which led to spontaneous self-formation of NE and SE areas across the plate [38, 56]. We observed that in mzOPC cultures, multiple zones were usually round but also had elongated shapes that varied in size, and they were formed by 3–4 rings, in contrast to other SEAM protocols that produced round multizones with 3–5 different concentric rings, as they were started from single colonies [29, 35, 53]. The efficiency usually ranged from 60 to 120 multizones per well of a 6-well plate resulting in high number of multiocular organoids with no significant differences between both cell lines.

Following the formation of ocular tissue-specific areas within the mzOPC stage, the subsequent stages of ocular organoid development in 3D were driven by ocular structures self-refolding, which prevented its disruption and maintained cell–cell interactions, cell numbers and cell signaling [57]. It is particularly interesting to study the crosstalk between different eye structures. The composition of multiocular organoids was demonstrated to vary and create more complex structures formed by RPE-retina-cornea or simpler structures containing retina-RPE, retina-cornea or RPE-cornea. This suggests that communication among ocular cell types can occur through specialized surface cell interactions or by secreted factors, as we observed different maturation stages or an increased number of corneal cells (CK3+) in multiocular organoids compared to individual corneal organoids. Moreover, tissue interactions between NR and RPE or between conjunctiva and cornea may foster stem cell niches containing ciliary margin-like or limbal-like growth zones. Taken together, we now showed that self-organizing multiocular organoids may not only generate highly organized ocular structures, but also orchestrate native oculogenesis. Despite that, it is clear that multiocular organoids do not form a true eye or possess a properly localized RPE layer, nor do they contain relevant non-neuroectodermal lineages, such as mesoderm.

Within these complex ocular organoids, retinal tissue has been demonstrated to mimic native human retinal tissue spatiotemporal development in a way that cannot be observed in animal models [58]. During early eye development, retinal progenitors emerge from PAX6 + cells in the early eye-field, which in turn require *RAX* and *CHX10* to proliferate [59]. Likewise, retinal cells that differentiated as 2D monolayers in the mzOPC expressed important retinal markers (*PAX6, RAX, LHX2, SIX3,* and *SIX6*), and the retinal neuroepithelium self-organized to form NR structures [60]. These NR regions were composed of a heterogeneous mix of retinal cells containing each major retinal cell type that organized into laminated structures. Once lifted, the NR formed retinal organoids consisting of layered structures with the main photoreceptor subtypes, including photoreceptors with rhodopsin, L/M-opsin and S-opsin in the outer retina and ganglion cells in the inner part, which emulated many of the temporal and spatial characteristics of in vivo development [61]. During human retinal development, photoreceptor OS emerge and elongate at weeks 16–20 of gestation [48], coinciding with the emergence of OS in our retinal organoids observed at day 140 (week 20). The OS observed had very few or disorganized disk stacks, which was likely due to the immaturity of the organoid. Electron microscopy data showed that electron-dense ribbons surrounded by synaptic vesicles and synaptophysin and vGlut1 expression were present, confirming that there were synapses between retinal layers [62, 63]. These results show that our retinal organoids could develop some synaptic maturity at day 140. However, longer incubation periods of up to 200 days may be needed to enhance photoreceptor OS maturation [64].

For corneal organoids to form, the emergence of SE during the differentiation of hiPSCs into eye-field commitment was related to signals that regulate eye development [49, 65]. For instance, the transcription factor p63, especially its isoform ΔNp63α, has been linked to stemness and is highly expressed in the basal layers of the CE and in the limbus [66]. The presence of p63 + cells together with PAX6 in mzOPCs and organoids suggests they have a primitive epithelial surface [65] similar to those obtained from human limbal cells [15]. Inhibition of BMP4 during SEAM differentiation it has been shown that decreases the expression of p63 [65]; however, in contrast, the presence of noggin (inhibitor of BMP4) during the first 30 differentiation days in the current mzOPC cultures did not block p63 expression or the formation of surface ectoderm. Upon further differentiation, SE areas formed corneal-like organoids with a transparent epithelium that was filled with fluid and subsequently stroma and expressed proteins specific to the cornea, conjunctiva and limbus as previously described [38, 54]. Given the common developmental origin and a degree of structural similarity, both cornea and conjunctiva are transparent multilayer epithelia with some distinct features, such as cell layer thickness, type of cells, or the presence of mucins for the tear film [67], but it was not possible to distinguish corneal or conjunctival epithelia by eye. To further verify the maturation of the corneal organoids and distinguish between corneal and conjunctival epithelia, we confirmed the protein expression of CK3, AQP1 and N-Cad [47, 68]. We did observe a certain degree of stratification after 140 days, supporting the conclusion that these cells are able to mature toward terminally differentiated and stratifying corneal epithelial cells, which was more evident in multiocular organoids. Although we observed some corneal regions, organoids contained mostly limbal-conjunctival epithelium expressing CK5, CK19, Na+/K+-ATPase and p63, which have been previously established as putative markers [69]. Interestingly, conjunctival-predominant corneal organoids contained stratified columnar-type cells with interspersed goblet-like cells expressing mucin 1. Moreover, corneal organoids also contain mesenchymal cells in addition to ocular-specific cells [38, 54]. Mesenchymal cells might be derived from NC cells, rather than from mesoderm cells, and they appeared during differentiation. It is likely that NC-like cells come from cells that are proximal to the ectoderm, such as periocular NC, during eye development [5], which might be important for the development of organoids and epithelial growth. Indeed, VIM + keratocyte-like cells were found to produce stromal collagen types I and V, which are important for shaping corneal organoids, generating a subepithelial basement membrane-like structure that resembles the Bowman layer.

Previously, other studies generated RPE spheroids that differed from the RPE organoids obtained with this system [17, 70–73]. Compact RPE spheroids were obtained from ARPE19 or primary RPE cell aggregation that did not acquire apical-basolateral polarity or form Bruch’s membrane-line, collagen-filled or fluid-filled stroma, which did not recapitulate the native RPE epithelium. In contrast, our study and the study by Liu et al. [53] generated RPE organoids consist of an RPE monolayer on the surface, with microvilli on the apical side and deposits of collagen on the basolateral side, resembling Bruch’s membrane. RPE organoids acquired pigmentation over time and could be isolated and purified to obtain an RPE cell monolayer, which could be easily amplified and passaged; exhibiting a proper RPE phenotype and function, such as phagocytosis of the photoreceptor OS and production of VEGF and epithelium barrier [53]. This makes the RPE organoids useful for cell therapy either by single cell injections [41, 42] or by transplantation half of the RPE organoid, as they are already polarized and can be integrated in a similar manner as RPE in sheets or scaffolds [74], although it uses its own ECM as a support.

Organoids generated using our self-organization protocol displayed high complexity and contained different eye parts that are derived from the ectoderm germ layer. Although these tissues resemble those of the developing eye, whether ocular tissue can be guided to organize into its natural conformation to mimic the eye must still be determined. One limitation is that in this culture system, the organization of the different ocular tissues within the organoids was random and did not recreate the primitive eye conformation. This is probably due to the starting point of the differentiation in 2D and the technique to lift the ocular structures. But more important, the key limitation of this protocol is the lack of integrated vasculature or immune cells necessary to fully mimic adult ocular tissue composition [75]. The presence of these extra cell types in our multiocular organoids is not relevant to emulating early ocular development. Nevertheless, for later stages of development, these components are crucial, as multilineage communication is required for tissue development that contributes to tissue maturation [76, 77]. To solve this, several creative approaches are being developed to generate more advanced organoids [76–79]. Some strategies that have been recently utilized include the fusion of organoids that have grown apart, replacing the undefined Matrigel matrix with defined synthetic hydrogels and guided self-organization [80]. One further step to achieve even more complex and complete organoids is to incorporate blood vessels [81] and microglia [82] arising from the mesoderm. Indeed, vasculature or microglia (either by single-cell addition or by organoid assembly) has been already added to integrate within neural organoids while they formed [76, 77, 79, 82–84], which overcomes the aforementioned limitation.

## Conclusion

This study demonstrates that by allowing multiocular structures from hiPSCs to self-organize, it is possible to obtain complex 3D multicellular ocular organoids that comprise the retina, cornea, RPE and stroma. A better understanding of eye development will likely be gained using multiple ocular cell cultures that closely mimic human early ocular tissue organization rather than single cell/tissue subtypes. This protocol not only avoids the problem of limited cell-type differentiation but also enables the simultaneous generation of RPE, retinal and corneal organoids. The hiPSC-derived organoids show remarkable similarity to the human retina, RPE and cornea, which we believe will pave the way for generating disease models and personalized medicine initiatives, such as drug screening platforms and/or gene and cell therapies. Blindness can be caused by many factors, from genetic defects and injuries of the anterior segment of the eye, such as corneal scars or infections, conjunctivitis, cataracts or keratitis, to posterior segment diseases, such as age-related macular degeneration, diabetic retinopathy or glaucoma. The different ocular organoids developed in this study display potential for use in developmental biology studies, disease modeling, drug testing and personalized medicine.

## Supplementary Information

**Additional file 1**. “Supplementary Information” containing figures for the characterization of mzOPC and multiocular organoids: CB30 line (Figures S1, S4, S5, S7-S9) and FiPS line (Figures S2, S3, S6), tables for antibodies and primers (Tables S1 and S2) along with information for the characterization of hiPSC lines, TEER and POS Phagocytosis Assays with the corresponding references.

## Data Availability

Not applicable.

## Abbreviations

AQP1: Aquaporin 1
AP-2: Activating enhancer-binding protein 2-alpha
ATRA: All-trans retinoic acid
bFGF: Basic fibroblast growth factor
CD59: Membrane attack complex inhibition factor
CD140: Platelet derived growth factor receptor alpha
CE: Corneal epithelium
CHX10: VSX2: visual system homeobox 2
CK3: Cytokeratin 3
CK5: Cytokeratin 5
CK19: Cytokeratin 19
CK12: Cytokeratin 12
COL8A1: Collagen Type VIII Alpha 1 Chain
COL8A2: Collagen Type VIII Alpha 2 Chain
CRX: Cone-rod homeobox
DAPI: 4′,6-Diamidino-2-phenylindol
DIV: Days in vitro
DKK-1: Dickkopf-1
FITC: Fluorescein isothiocyanate
GAPDH: Glyceraldehyde 3-phosphate dehydrogenase
GCL: Ganglion cell layer
GFAP: Glial fibrillary acidic protein
hESC: Human embryonic stem cells
hiPSC: Human induced pluripotent stem cells
IGF-1: Insulin growth factor 1
INL: Inner nuclear layer
IS/OS: Inner and outer segments
L: Lens
mzOPC: Multizone ocular progenitor cells
NC: Neural crest
N-CAD: N-cadherin
NE: Neuroectoderm
NR: Neuroretina
NRL: Neural Retina Leucine Zipper
MITF: Microphthalmia-associated transcription factor
Na+/K+-ATPase: Sodium/potassium-transporting ATPase
NR2E3: Nuclear receptor subfamily 2 group E member 3
OCT4: Octamer-binding transcription factor 4
ONL: Outer nuclear layer
OPL: Outer plexiform layer
P63: Delta-N tumor protein p63
P75-NGFR: Nerve growth factor receptor
PAX3: Paired box 3
PAX6: Paired box 6
PE: Pigmented epithelium
PKC-alpha: Protein kinase C
POS: Photoreceptor outer segment
qPCR: Quantitative polymerase chain reaction
RAX: Retinal homeobox protein Rx
RECOV: Recoverin
RPE: Retinal pigment epithelium
SD: Standard deviation
SE: Surface ectoderm
SEAM: Self-formed ectodermal autonomous multizone
SIX6: SIX homeobox 6
SOX2: SRY-Box transcription factor 2
SOX9: SRY-Box transcription factor 9
SOX10: SRY-Box transcription factor 10
SSEA3: Stage-specific embryonic antigen 3
SSEA4: Stage-specific embryonic antigen 4
TEER: Transepithelial electrical resistance
SYNAP: Synaptophysin
Tra-1–60: T cell receptor alpha locus
TFAP2A: Transcription factor AP-2 alpha
TUJ1: Beta-III tubulin
vGLUT1: Vesicular glutamate transporter 1
ZO-1: Zonula occludens
