# Supplementary material for: Multiocular organoids from human induced pluripotent stem cells displayed retinal, corneal, and retinal pigment epithelium lineages
Source: Stem Cell Res Ther. 2021 Nov 22;12:581. doi: 10.1186/s13287-021-02651-9 (PMC8607587; doi:10.1186/s13287-021-02651-9)
Supplement: Supplementary file 1 — Additional file 1. “Supplementary Information” containing figures for the characterization of mzOPC and multiocular organoids: CB30 line (Figures S1, S4, S5, S7-S9) and FiPS line (Figures S2, S3, S6), tables for antibodies and primers (Tables S1 and S2) along with information for the characterization of hiPSC lines, TEER and POS Phagocytosis Assays with the corresponding references. [file 13287_2021_2651_MOESM1_ESM.pdf]

ADDITIONAL FILE

Multiocular organoids from human induced pluripotent stem cells displayed retinal, corneal, and retinal pigment epithelium lineages

Helena Isla-Magrané, Anna Veiga, José García-Arumí, Anna Duarri*

Tables: 2

Figures: 9

METHODS

Characterization of hiPSC lines

CBiPS30-4F-5 and FiPS-4F-7 cell lines were fully characterized and banked into the Spanish National Stem Cell Bank/ Banco Nacional de Líneas Celulares (BNLC). This information can be found at the website:

https://www.isciii.es/QueHacemos/Servicios/BIOBANCOS/BNLC/Paginas/LineasiPS.aspx

Transepithelial Electrical Resistance Measurements

Human iPSC and RPE cells were seeded on Matrigel-coated transwells filter inserts (Millipore, USA) and cultured until the complete formation of the cell monolayer. hiPSC were cultured in mTSeR1 (StemCell Technologies, Vancouver, BC, Canada) and RPE cells were cultured in IM medium consisting of Dulbecco's Modified Eagle´s Medium/Nutrient Mixture F-12 (DMEM/F12), 5% fetal bovine serum, 0.1 mM non-essential amino acids, 2 mM GlutaMax, 1% N2, 1% B27 (all the previous reagents were from Gibco, Thermo Fisher Scientific, Inc., Waltham, MA, USA), 10 mM β-glycerol phosphate (Sigma Aldrich, St. Louis, MO, USA), recombinant human IGF1 (10 ng/ml; R&D Systems, Minneapolis, MN, USA) and 10 mM nicotinamide (Sigma Aldrich, St. Louis, MO, USA) Electrical measurements were performed every 5 days for 3 weeks in biological triplicates using an epithelial Volt/Ohm Meter (EVOM3; World Precision Instruments Inc., USA). Transepithelial electrical resistance (TEER) values were corrected by subtracting values of a blank and multiplied by the cell growth area. Values were presented in Ω·cm2**.**

Photoreceptor Outer Segment Phagocytosis Assays

RPE phagocytosis activity was assessed by analyzing the binding and internalization of photoreceptor outer segments (POS) labeled with fluorescein isothiocyanate (FITC), as described previously [1]. RPE cells were incubated with POS-FITC for 12 hours at 37ºC with 5% (v/v) CO2 in IM medium, washed four times in phosphate-buffered saline with Ca2+ and Mg2+ and then fixed in 4% (w/v) paraformaldehyde for 20 minutes at room temperature. Immunocytochemistry was performed as described [2] using described in Table S1 and phalloidin-Alexa 647(1:200; Invitrogen). Confocal images were taken with a TCS SP5 confocal microscopy (Leica Microsystems, Wetzlar, Germany).

References

1 Nandrot EF, Anand M, Almeida D, et al. Essential role for MFG-E8 as ligand for αvβ5 integrin in diurnal retinal phagocytosis. Proc Natl Acad Sci U S A 2007;104:12005–12010.

2 Salas A, Duarri A, Fontrodona L, et al. Cell therapy with human induced pluripotent stem cell-derived retinal pigment epithelium and retinal precursor cells prevents visual function loss in a rat model of retinal degeneration. Molecular Therapy - Methods & Clinical Development 2021;0.

Table S1: List of primary antibodies

Antibody	Supplier and reference	Species	Dilution
**Actin**	MP Biomedicals, 691001	Mouse	1:1000
**Aquaporin-1**	Santa Cruz Biotechnology, sc-25287	Mouse	1:50
**AP2**	Santa Cruz, sc-184	Mouse	1:100
**Bestrophin 1**	Santa Cruz Biotechnology, sc-32792	Mouse	1:25
**B- Opsin**	Millipore, AB5407	Rabbit	1:100
**Calbindin**	Swant, 300	Mouse	1:50
**CHX10**	Abcam, ab16141	Sheep	1:100
**Collagen I**	Abcam, ab34710	Rabbit	1:100
**Collagen IV**	Abcam, ab6586	Rabbit	1:100
**CRX**	Abnova, H00001406-M02	Mouse	1:50
**Cytokeratin 19**	Genetex, GTX112666	Rabbit	1:100
**Cytokeratin 19**	Santa Cruz Biotechnology, sc-374192	Mouse	1:100
**Cytokeratin 19**	Santa Cruz Biotechnology, sc- 33119	Goat	1:100
**Cytokeratin 3/2p**	Santa Cruz Biotechnology, sc-80000	Mouse	1:100
**Cytokeratin 5**	Abcam, ab24647-50	Rabbit	1:100
**GFAP**	Dako, Z0334	Rabbit	1:1000
**Ki67**	Abcam, AB16667	Rabbit	1:100
**MITF**	Santa Cruz Biotechnology, sc-56725	Mouse	1:25
**Na+/K+-ATPase**	Millipore, 05-369	Mouse	1:50
**NANOG**	R&D Systems, AF1997	Goat	1:25
**NRL**	R&D Systems, AF2945	Goat	1:25
**OCT4**	Santa Cruz, sc-5279	Mouse	1:25
**P63**	Abcam, ab735	Mouse	1:50
**PAX6**	Covance, PRB278P	Rabbit	1:100
**PKCα**	Santa Cruz, sc-8393	Mouse	1:100
**RAX**	Abcam, ab23340	Rabbit	1:50
**Recoverin**	Millipore, AB5585	Rabbit	1:500
**RG-Opsin**	Millipore, AB5405	Rabbit	1:100
**Rhodopsin**	Sigma, O4886	Mouse	1:500
**p75 NGFR**	Santa Cruz Biotechnology, sc- 271708	Mouse	1:50
**Pericentrin**	Abcam, ab28144	Mouse	1:50
**RPE65**	Santa Cruz Biotechnology, sc-73616	Mouse	1:100
**SOX2**	Santa Cruz Biotechnology, sc-17320	Goat	1:25
**SOX9**	Invitrogen, 711048	Rabbit	1:200
**SOX10**	Santa Cruz Biotechnology, sc-365692	Mouse	1:50
**SSEA1**	Iowa, MC-480	Mouse	1:2
**SSEA3**	Iowa, MC-631	Rat	1:2
**SSEA4**	Iowa, MC-813-70	Mouse	1:2
**Synaptophysin**	Millipore, MAB329-C	Mouse	1:100
**TRA-1-60**	Chemicon, MAB4360	Mouse	1:100
**TRA-1-81**	Chemicon, MAB4381	Mouse	1:100
**Tuj1**	Covance, MMS-435P	Mouse	1:1000
**vGUT1**	Millipore, AB5905	Guinea pig	1:50
**Vimentin**	Cell signaling kit Arigobio, SQab1721	Rabbit	1:100
**ZO-1**	Millipore, ab2272	Rabbit	1:100
**γ-cristallin**	Santa Cruz Biotechnology, sc-22415	Goat	1:50

Table S2: List of primers used in PCR and RT-qPCR

Gene	Forward	Reverse
**AQP1**	ACCTCCTGGCTATTGACTACA	CCCTTCTATTTGGGCTTCATCT
**CHX10**	GGCGACACAGGACAATCTTTA	TTCCGGCAGCTCCGTTTTC
**CK12**	AGCAGAATCGGAAGGACGCTG	ACCTCGCTCTTGCTGGACTGAAA
**CK19**	ACAGCCACTACTACACGACC	CCTGTTCCGTCTCAAACTTGGT
**CK3**	ACGTGACTACCAGGAGCTGATG	ATGCTGACAGCACTCGGACACT
**COL8A1**	CCTGGGTCAGCAAGTACCTC	TTGTTCCCCTCGTAAACTGG
**COL8A2**	ATGCTGGGGACTCTGACAC	GGTAGAGGCATTTCCAGGTACT
**CRX**	TCCAGGGTTCAGGTTTGGTT	CATCTGTGGAGGGTCTTGGG
**GAPDH**	CCTGCACCACCAACTGCTTAG	TGGCATGGACTGTGGTCATG
**LHX2**	ATGCTGTTCCACAGTCTGTCG	GCATGGTCGTCTCGGTGTC
**MITF**	GTGCCAACTTCTTTCATCA	ACCTAAACCGTCCATTCA
**Na+/K+- ATPase**	CAGGGCAGTGTTTCAGGCTAA	TCGACGATTTTGGCGTATCTT
**NR2E3**	GGCTTCTTCAAGAGGAGCGT	CGGGACTCAGTGTTGGACTC
**OCT3/4**	GTTCTTCATTCACTAAGGAAGG	CAAGAGCATCATTGAACTTCAC
**P63**	GAAAACAATGCCCAGACTCAATTT	TCTGCGCGTGGTCTGTGTTAT
**PAX3**	CCACAAGATCGTGGAGATGG	ACCGCGTCCTTGAGTAATTT
**PAX6**	TCTAATCGAAGGGCCAAATG	TGTGAGGGCTGTGTCTGTTC
**RAX**	GCGAAGCGAAACTGTCAGAG	TTCTGGAACCACACCTGGACC
**RECOVERIN**	TCTACGACGTGGACGGTAACG	CGTCCTCGGGAGTGATCATT
**RHODOPSIN**	GGGAGAACCATGCCATCAT	TCGTCTCCGTCTTGGA
**SIX3**	CACTCCCACACAAGTAGGCA	GCTGGAGGTTACCGAGAGGA
**SIX6**	ACGGCGAACAGAAGACACAC	TGCTGGAGTCTGTTCTTGGCT
**SOX9**	TGAAGAAGGAGAGCGAGGA	CGCGGCTGGTACTTGTAAT
**TFAP2**	GTCTCCGCCATCCCTATTAAC	GGACAGCTTCTCCCTCTACTA

FIGURE S1

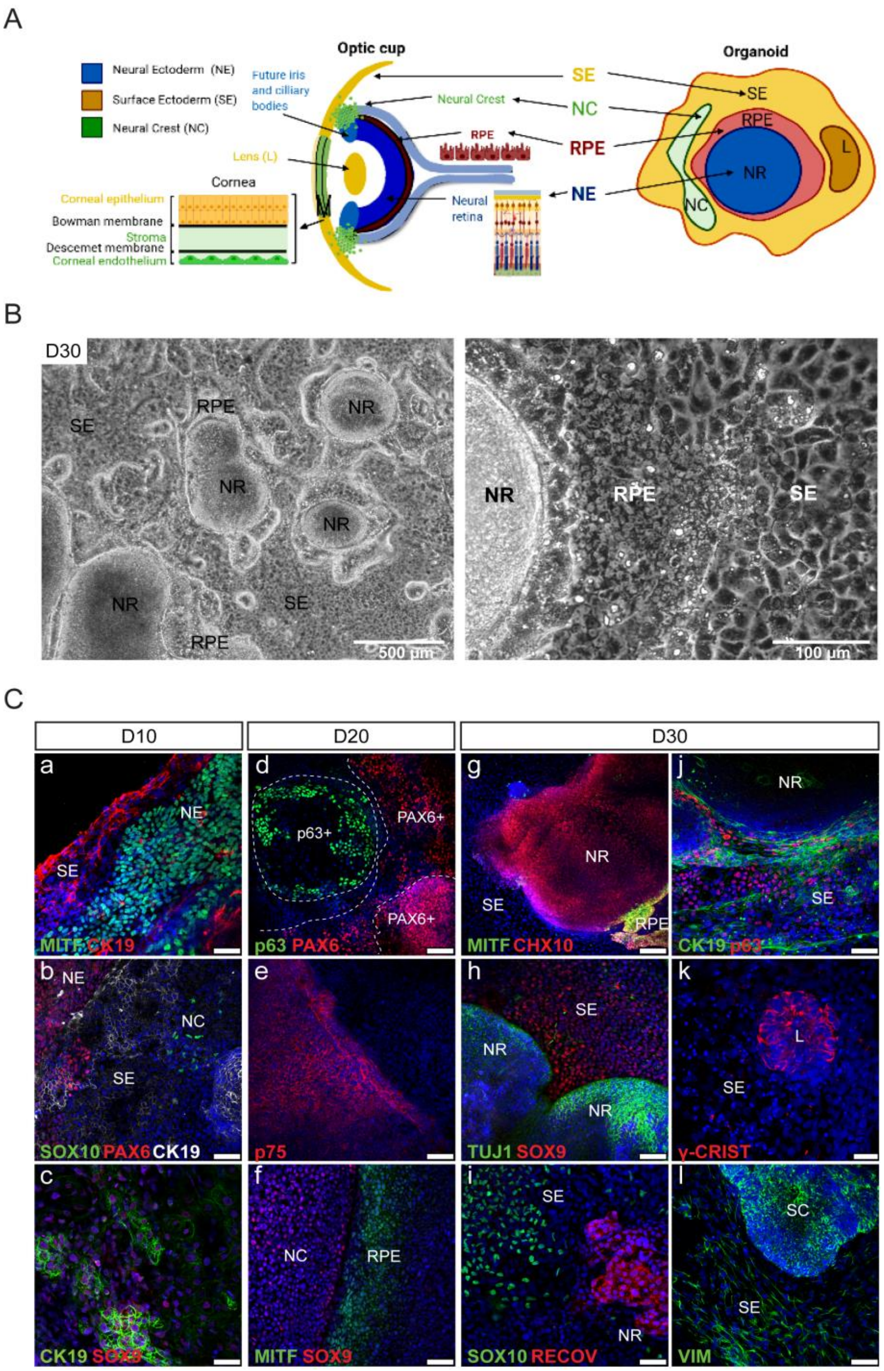

Figure S1: Generation of multizone ocular progenitor cells from CB30 hiPSC line. A) Schematic diagram of multizone ocular progenitor cells (mzOPCs) differentiation showing different ectodermal lineages mimicking eye development. B) Representative phase-contrast image of mzOPCs at day 30 of differentiation containing regions of surface ectoderm (SE), retinal pigment epithelium (RPE), and neural retina (NR). Scale bar: 500 µm; 100 µm. C) Immunofluorescence images of mzOPCs at days 10, 20, and 30 of differentiation presenting areas of neuroectoderm (NE), SE, RPE, NR, neural crest (NC), lentoid cell cluster (L), and stromal cells (SC), immunolabelled with PAX6 (neuroectoderm marker); TUJ1 and NRL (NR markers); MITF (RPE marker); CK19 and p63 (SE markers); SOX9, SOX10, and p75-NGFR (NC markers); γ-crystallin (lens marker); and vimentin (VIM) (SC marker). Nuclei were stained in DAPI. Scale bars: 100 µm in d, g; 50 µm in b, e, f, h, j, l;.25 µm in a, c, I, k.

FIGURE S2

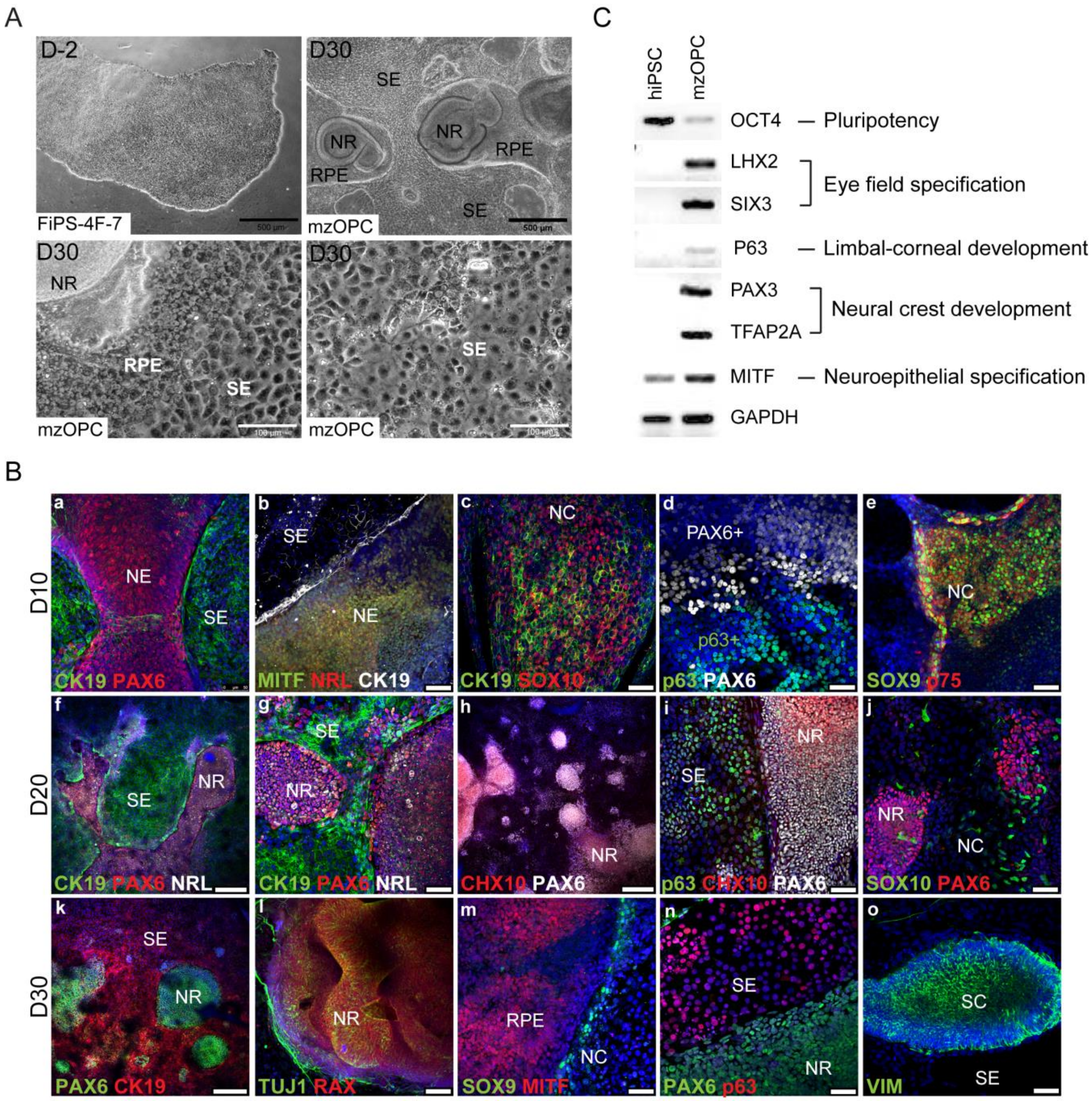

Figure S2: Generation of multizone ocular progenitor cells from FiPS hiPSC line. A) Phase-contrast images of human induced pluripotent stem cells (hiPSC) at day -2 showing a compact stem cell colony, and multizone ocular progenitor cells (mzOPCs) after 30 days of differentiation showing different ocular lineages: neuroretina (NR), retinal epithelium (RPE), and surface ectoderm (SE). Scale bars: 500 µm; 100 µm. **B)** Immunocytochemistry of mzOPCs at days 10, 20, and 30 of differentiation showing neuroectoderm (NE), NR, RPE, SE, neural crest cells (NC), and stromal cells (SC). Cells were stained with antibodies against PAX6 (NE marker), MITF (RPE marker), NRL, RAX, CHX10, and TUJ1 (NR markers), p63 and CK19 (SE markers), SOX9, SOX10, and p75-NGFR (NC markers), and VIM (stromal fibroblasts marker). Nuclei are stained with DAPI. Scale bars: 250 µm in f, h, k; 50 µm in b-d, g, I, j, l-o. **C)** PCR analysis of specific eye-field, NE, SE, RPE, and neural crest (NC) gene expression levels in samples from hiPSC and mzOPCs at day 30 of differentiation. Expression of *GAPDH* was used as an internal control.

FIGURE S3

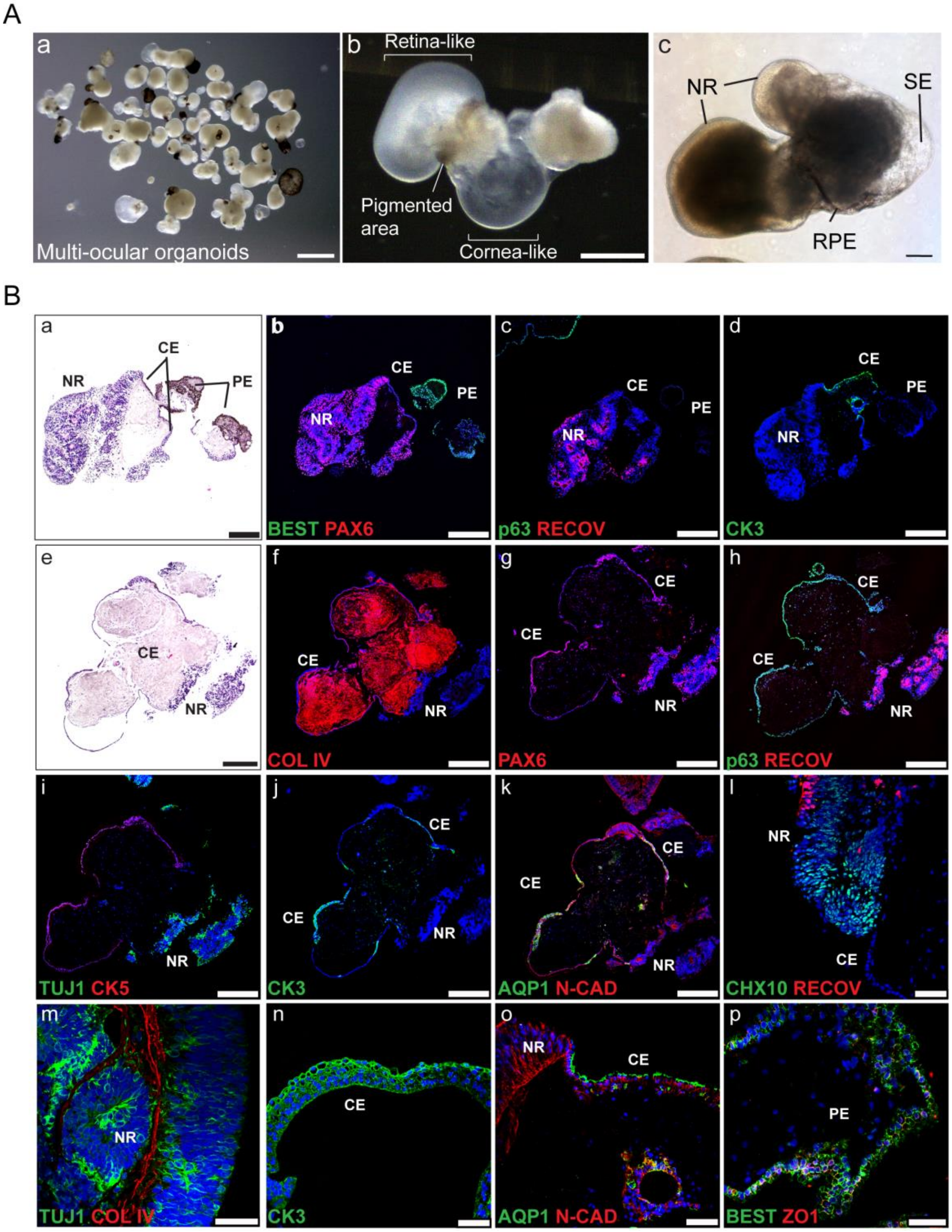

Figure S3: Generation of multiocular organoids from FiPS hiPSC line. A) (a) Microscopic image of a mixed multiocular organoids culture at day 90. (b, c) Bright-field image of multiocular organoid showing different ocular regions: neuroretina (NR), pigmented epithelium (PE), and surface ectoderm (SE). Scale bars: 2 mm in a; 500 µm in b; 150 µm in c. B) Hematoxylin and eosin (HE) staining (a, e) and immunofluorescence images of the multiocular organoid paraffin section with different ocular structures. Sections are labeled with specific antibodies PAX6, RECOV, and TUJ1 (NR markers); CK3, p63, CK5, AQP1, N-CAD (corneal-conjunctival epithelium markers); and BEST and ZO1 (RPE markers). The stroma is stained with collagen IV (COL IV). Nuclei are stained with DAPI. Scale bars: 250 µm in a-k; 50 µm in l-p.

FIGURE S4

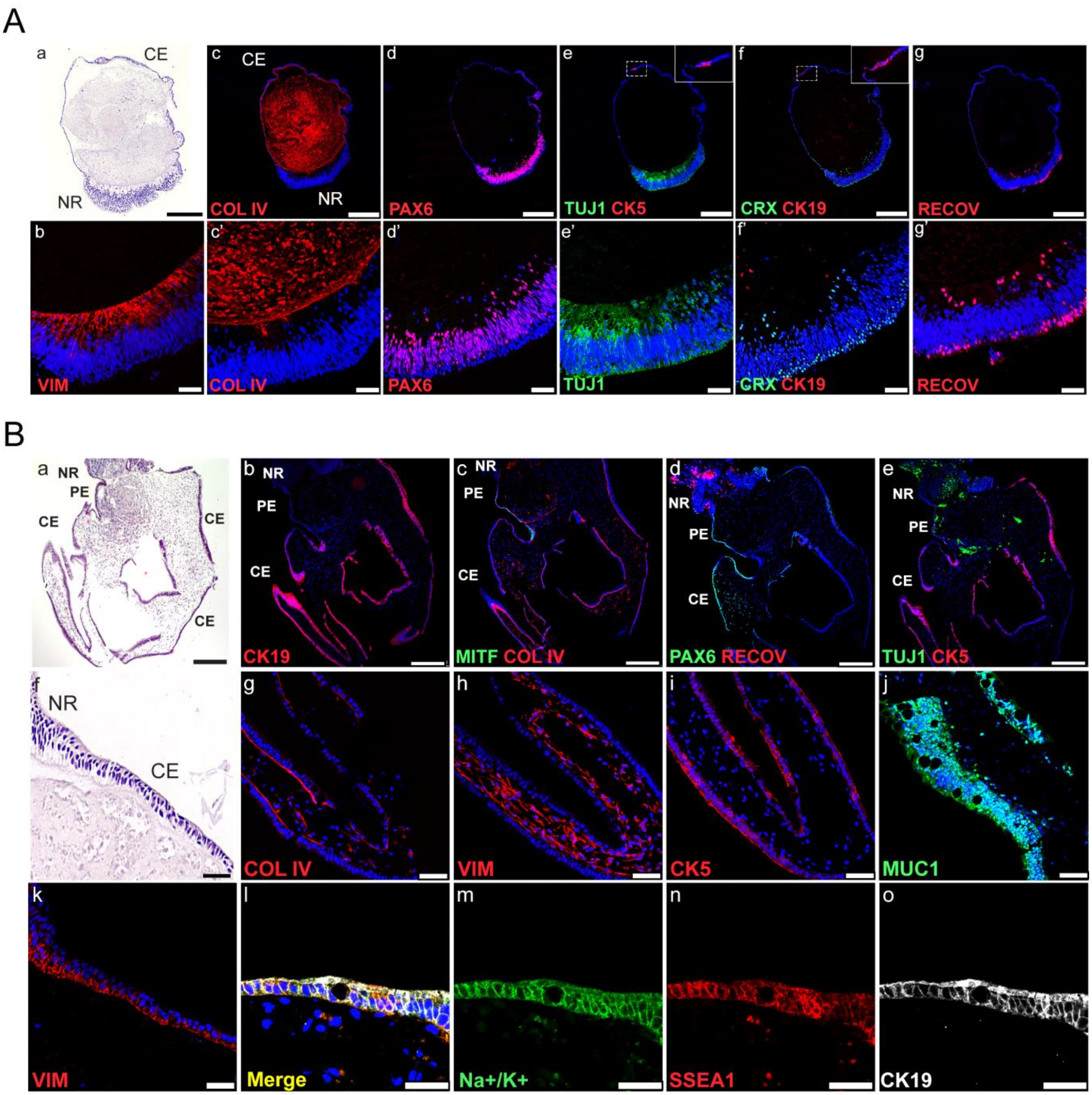

Figure S4: CB30-derived multiocular organoids composed of retinal, corneal, and pigmented areas. A) Hematoxylin and eosin (HE) staining of multiocular organoid paraffin section formed by neuroretina (NR) and corneal epithelium (CE) (a). Immunofluorescent images showing expression of collagen IV (COL IV) and vimentin (VIM; b, I, m, n) in the stroma of the organoid; PAX6 (d, d') and TUJ1 (e, e') in the inner retina, and CRX (f, f') and recoverin (RECOV; g, g') in the outer retina; CK5 (e) and CK19 (f) is detected in the corneal-conjunctival epithelium. Scale bars: 250 µm in a, c-g; 50 µm in b, c'-g'. B) HE (a, f) and immunofluorescent images of multiocular organoid paraffin section formed by NR, CE, and pigmented epithelium (PE). Corneal-conjunctival epithelium is stained with CK19 (b, o), CK5 (e, i), MUC1 (mucin-1, j), Na+/K+-ATPase (m) and SSEA1 (n). Stroma is stained with COL IV (c, g) and VIM (h, k). PE is stained with MITF (c) and PAX6 (d). Neuroretina is stained with RECOV (d), TUJ1 (e). Nuclei are stained in DAPI. Scale bars: 250 µm in a-e; 50 µm in f-k; 25 µm in l-o.

FIGURE S5

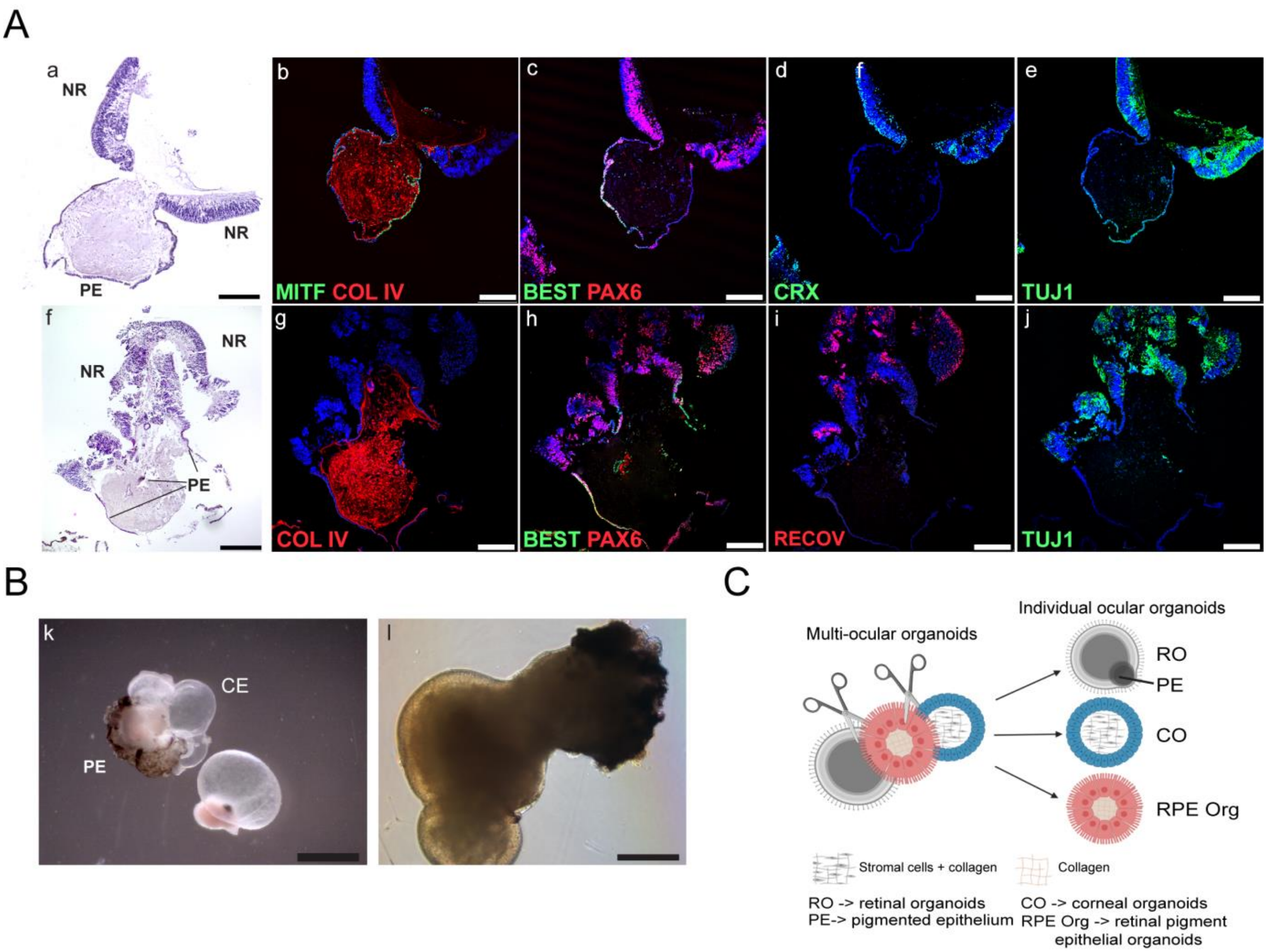

Figure S5: CB30-derived multiocular organoids composed by retinal and pigmented areas. A) Hematoxylin and eosin staining (a,f) and immunohistochemistry images of multiocular organoids exhibiting retinal (CRX, TUJ1, recoverin (RECOV), and PAX6), and RPE regions (MTIF, BEST, and PAX6) filled with collagen type IV (COL IV) (b-e; g-j). Scale bars: 250 µm. B) Bright-field images of multiocular organoids composed of the pigmented epithelium (PE) and the cornea (k), and the PE and the retina (l). Scale bars: 500 µm (k); 150 µm (l). C) Schematic illustration of isolation of different ocular regions from multiocular organoids to generate retinal organoids (RO) and RO containing pigmented areas (RO+PE); corneal organoids (CO) containing stromal cells and collage, and CO containing pigmented areas (CO+PE); and RPE organoids containing collagen.

FIGURE S6

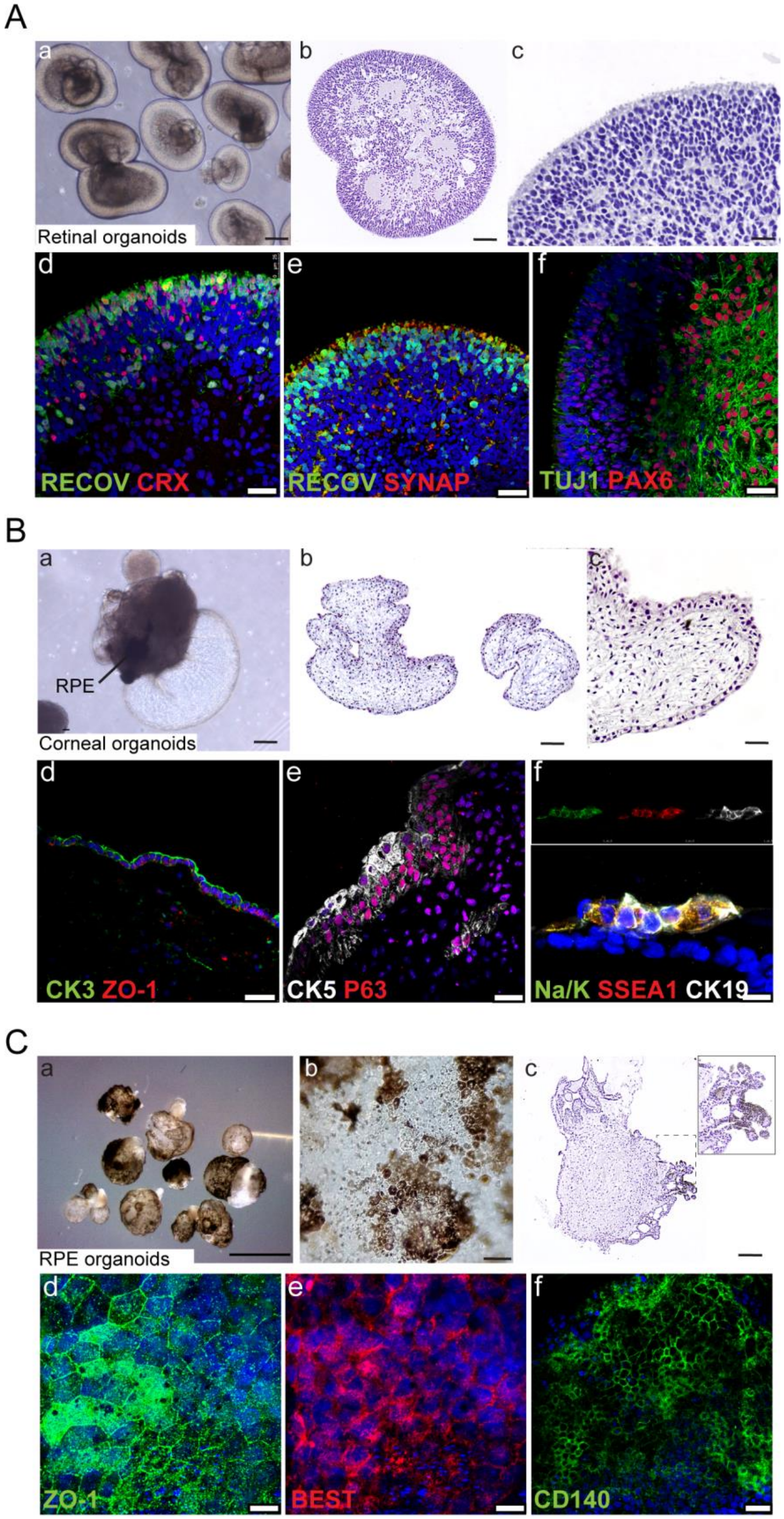

Figure S6: FiPS-derived individual retinal, corneal, and RPE organoids. A) Bright-field image of retinal organoids in suspension culture at day 140 (a) showing laminated neuroretinas (NR). Hematoxylin and eosin

staining (HE) staining images of retinal organoid paraffin sections (b, c) showing stratified NR at the apical site. Immunofluorescence images of retinal organoid paraffin sections (d-f) stained with specific antibodies against recoverin (RECOV) and CRX (photoreceptor markers), synaptophysin (SYNAP; presynaptic marker); PAX6 (bipolar and ganglion cell marker), and tubulin beta III (TUJ1; ganglion cell marker). Scale bars: 300 µm (a); 100 µm (b); 25 µm (c-f). B) Bright-field image of corneal-like organoids in suspension culture at day 140 in all-trans retinoic acid (a) showing spherical and transparent organoid corresponding to corneal-conjunctival epithelium. Some corneal organoids present a pigment epithelium (RPE) region on one side. HE staining images of corneal organoid paraffin sections (b, c) showing stratified epithelium in the apical side fulfilled with stromal-like cells. (d-f) Immunofluorescence images of corneal-conjunctival epithelium stained with cytokeratin 3 (CK3) and zonula occludens (ZO-1) (d), CK5 and p63 (e) and CK19, Na+/K+-ATPase (Na/K), and SSEA1 (f). Scale bars: 300 µm (a); 100 µm (b); 50 µm (c, d); 25 µm (e); 10 µm (f). C) Bright-field image of RPE organoids in suspension culture at day 140 (a) and magnification of the organoid apical surface (b) showing cuboidal, pigmented epithelial cells. HE staining image of retinal organoid paraffin sections (c) showing pigmented epithelium on the surface and lumen filled with stroma. Insets: magnification of the region of interest (dashed square). (d-f) Immunofluorescence images of RPE organoid surface stained with ZO-1, bestrophin1 (BEST), and CD140 antibodies. Scale bars: 2 mm (a); 100 µm (c); 25 µm (b, f); 10 µm (d, e). Nuclei are stained with DAPI.

FIGURE S7

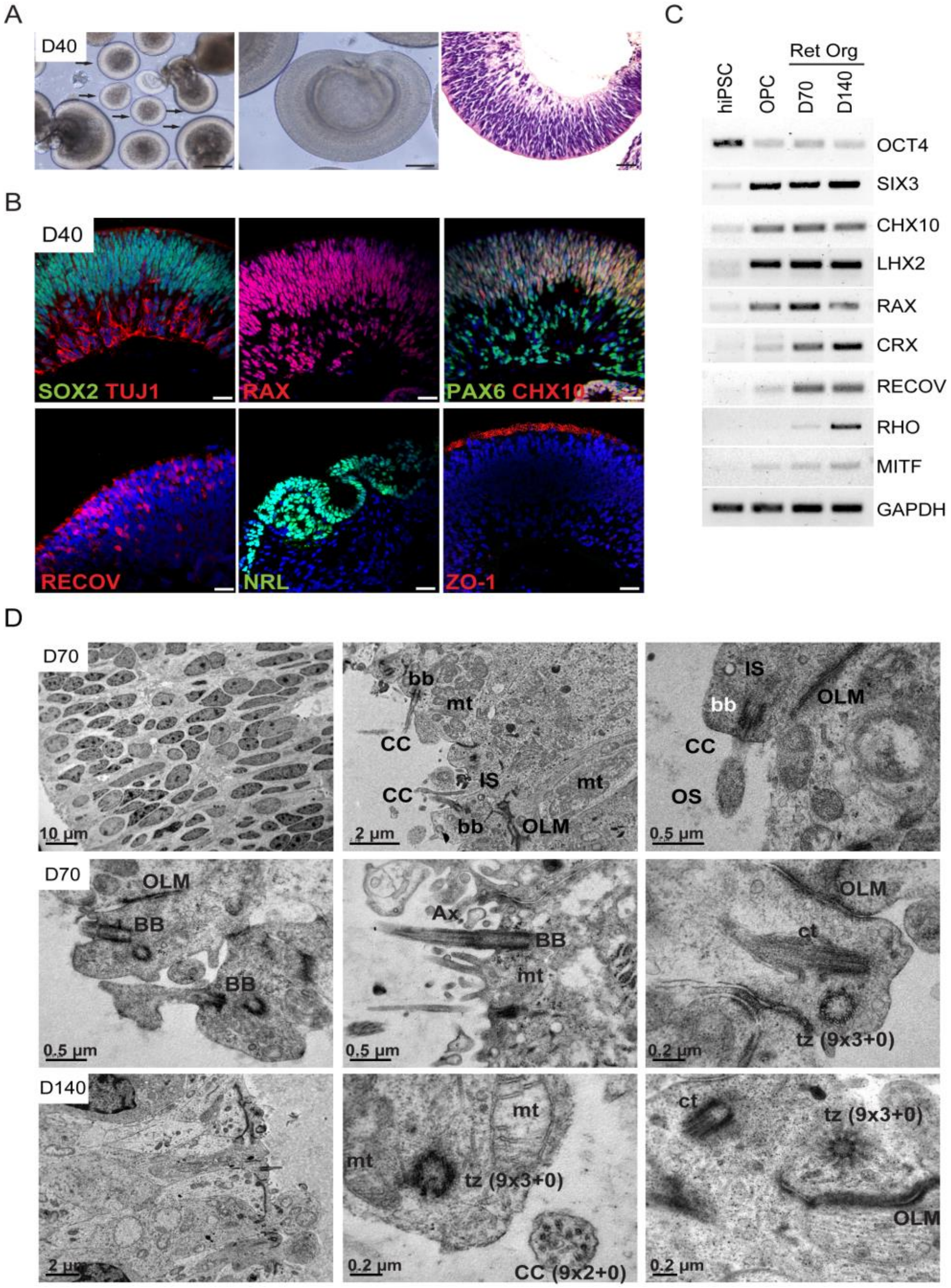

Figure S7: Characterization of retinal organoids generated from CB30 hiPSC line. A) Bright-field images of retinal organoids derived from hiPSC at day 40 of differentiation. Arrows indicate developing laminated retinal tissue. (Right) Hematoxylin and eosin staining of a paraffin section showing lamination of retinal tissue. Scale bars: 300 µm (left); 100 µm (middle); 50 µm (right). B) Confocal images of retinal organoid paraffin sections at day 40 immunostained with SOX2, TUJ1, PAX6, CHX10, RECOV, NRL, and ZO-1. Scale bars: 25 µm. C) PCR analysis of specific eye-field, retinal, and retinal pigment epithelium gene expression levels in samples from human induced pluripotent stem cells (hiPSC), ocular progenitor cells (OPC) at day 30 of differentiation, and retinal organoids (Ret Org) at days 70 and 140 in culture. Expression of *GAPDH* was used as an internal control. D) Transmission electron microscopy analysis of retinal organoids at days 70 and 140 show several retinal structures of connecting cilia (CC), basal bodies (bb), centriole (ct) inner segments (IS), mitochondria (mt), outer limiting membrane (OLM), outer segments (OS), the classical array of nine triplet and doublet microtubules of bb and transition zone (tz) of the CC, respectively. Scale bars are indicated in the images.

FIGURE S8

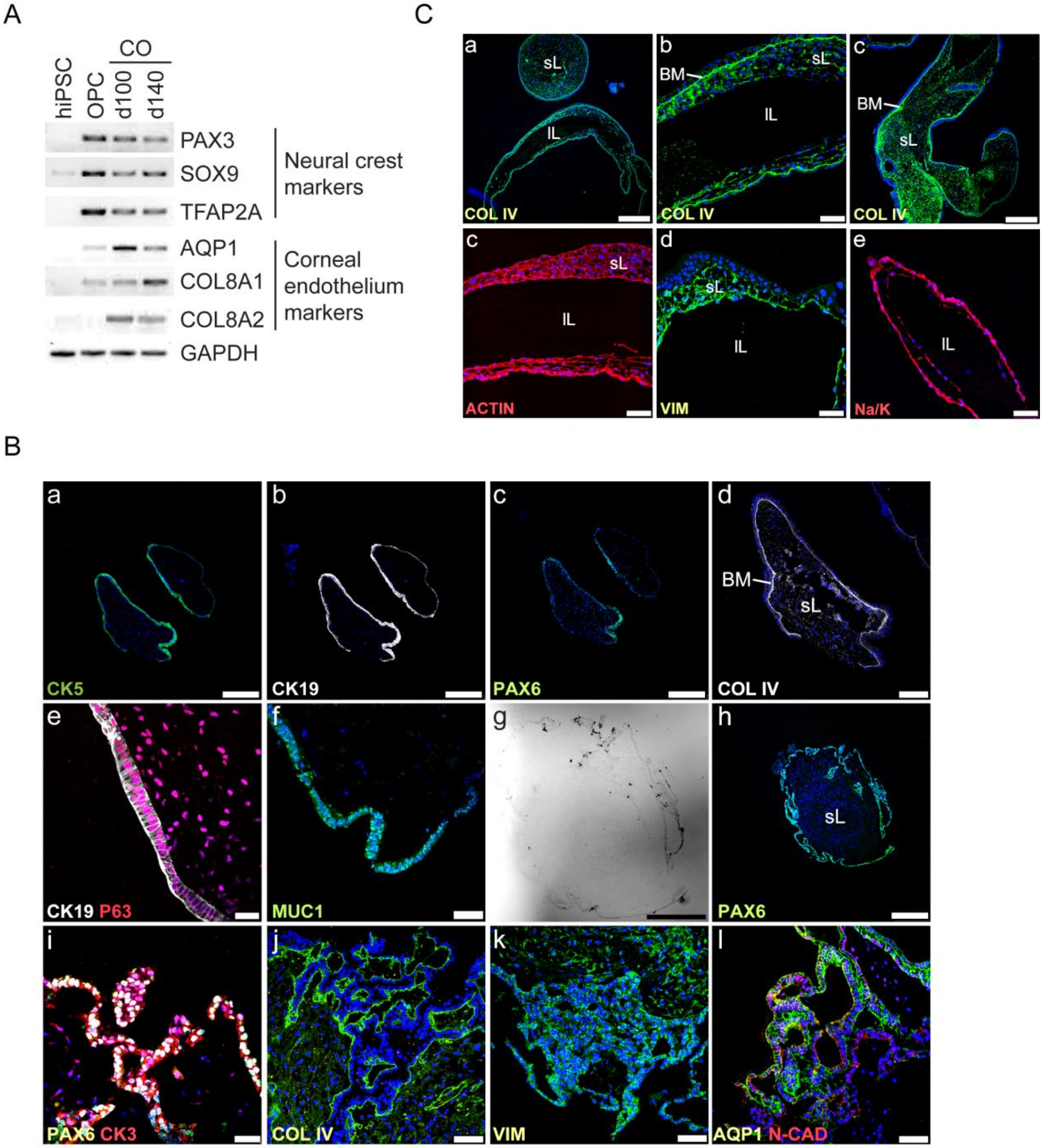

Figure S8. Characterization of corneal organoids derived from CB30 hiPSC line. A) PCR analysis of neural crest progenitors and corneal endothelium gene expression in samples from human induced pluripotent stem cells (hiPSC), ocular progenitor cells (OPC) at day 30 of differentiation, and corneal organoids (CO) at days 100 and 140 in culture. Expression of *GAPDH* was used as an internal control. B) Confocal images of corneal organoids at day 140 paraffin sections immunolabelled with cytokeratin 3, 5, and 19 (CK3, CK5, CK19), PAX6, p63, mucin 1 (MUC1), collagen type IV (COL IV), vimentin (VIM), aquaporin-1 (AQP1) and N-cadherin (N-CAD). Lumen was filled with stroma (sL). Pigmented cells are shown in the bright-field image (g). Scale bars: 250 µm in a-c, g, h; 100 µm in d; 50 µm in f, j-l; 25 µm in e, i, C) Immunofluorescence images of corneal endothelial-like organoids with a stromal lumen (sL) and fluid-filled lumen (lL) stained with COL IV (forming a basement membrane (BM) similar to Descemet's membrane), actin, VIM and Na+/K+-ATPase (Na/K). Scale bars: 250 µm in a, c; 50 µm in b, d-f. Nuclei are stained with DAPI.

FIGURE S9

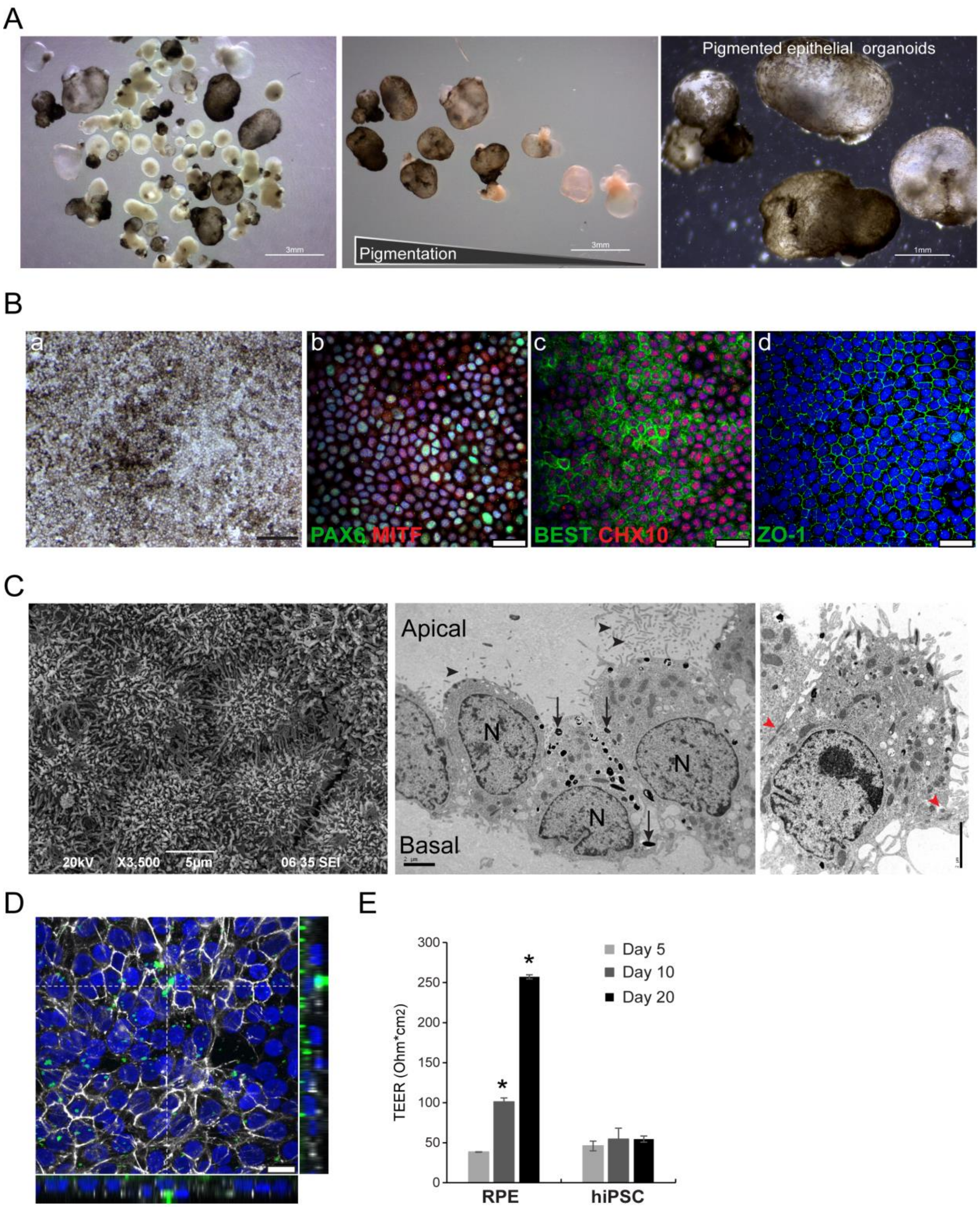

Figure S9. Pigmentation of retinal pigment epithelial (RPE) organoids and derivation of RPE cell culture. A) Bright-field images of CB30 hiPSC-derived RPE organoids in suspension at day 120 cultured without *all-trans* retinoic acid from day 90. Scale bars: 3 mm in a-b; 1 mm in c. B) (a) Phase-contrast image of RPE organoid-derived RPE cells cultured in monolayers after cell expansion. Scale bar: 100 μm. (b-c) Immunofluorescence staining of RPE cell culture with BEST, ZO-1, PAX6, MITF, RPE65, and CHX10. Scale bars: 25 μm. C) (Left) Scanning electron microscopy image of the RPE cell culture. RPE apical cell surface shows hexagonal cell

morphology and apical microvilli. Scale bar: 5 µm. (Right) Transmission electron microscopy image of an RPE cell monolayer containing melanosomes (arrows), basal location of nuclei (N), apical microvilli (arrowheads), and tight junctions (red arrowheads). Scale bar: 2 µm. D*) In vitro* phagocytosis assay with FITC-labelled photoreceptor outer segments (POS). Z-stack fluorescent images with orthogonal views showed the location of internalized FITC-POS in RPE cells. Tight junctions were stained with ZO-1 (white) to show the apical side. Scale bar: 10 µm. Nuclei are stained with DAPI. E) Graph showing transepithelial electrical resistance (TEER) for hiPSC and RPE cell cultured in transwells (n = 3) at indicated days in culture. Data are expressed as mean ± SD, * p<0,01 using unpaired Student's t-test.
